# Developing a ceRNA-based lncRNA-miRNA-mRNA regulatory network to uncover roles in skeletal muscle development

**DOI:** 10.3389/fbinf.2024.1494717

**Published:** 2025-01-15

**Authors:** Wang Wenlun, Yu Chaohang, Huang Yan, Li Wenbin, Zhou Nanqing, Hu Qianmin, Wu Shengcai, Yuan Qing, Yu Shirui, Zhang Feng, Zhu Lingyun

**Affiliations:** ^1^ Department of Food Science and Engineering, Moutai Institute, Renhuai, Guizhou, China; ^2^ Department of Biology and Chemistry, College of Sciences, National University of Defense Technology, Changsha, Hunan, China

**Keywords:** lncRNA, muscle development and regeneration, cell proliferation and differentiation, muscle atrophy, ceRNA network, signaling pathway

## Abstract

The precise role of lncRNAs in skeletal muscle development and atrophy remain elusive. We conducted a bioinformatic analysis of 26 GEO datasets from mouse studies, encompassing embryonic development, postnatal growth, regeneration, cell proliferation, and differentiation, using R and relevant packages (limma et al.). LncRNA-miRNA relationships were predicted using miRcode and lncBaseV2, with miRNA-mRNA pairs identified via miRcode, miRDB, and Targetscan7. Based on the ceRNA theory, we constructed and visualized the lncRNA-miRNA-mRNA regulatory network using ggalluvial among other R packages. GO, Reactome, KEGG, and GSEA explored interactions in muscle development and regeneration. We identified five candidate lncRNAs (Xist, Gas5, Pvt1, Airn, and Meg3) as potential mediators in these processes and microgravity-induced muscle wasting. Additionally, we created a detailed lncRNA-miRNA-mRNA regulatory network, including interactions such as lncRNA Xist/miR-126/IRS1, lncRNA Xist/miR-486-5p/GAB2, lncRNA Pvt1/miR-148/RAB34, and lncRNA Gas5/miR-455-5p/SOCS3. Significant signaling pathway changes (PI3K/Akt, MAPK, NF-κB, cell cycle, AMPK, Hippo, and cAMP) were observed during muscle development, regeneration, and atrophy. Despite bioinformatics challenges, our research underscores the significant roles of lncRNAs in muscle protein synthesis, degradation, cell proliferation, differentiation, function, and metabolism under both normal and microgravity conditions. This study offers new insights into the molecular mechanisms governing skeletal muscle development and regeneration.

## 1 Introduction

**FIGURE 1:**
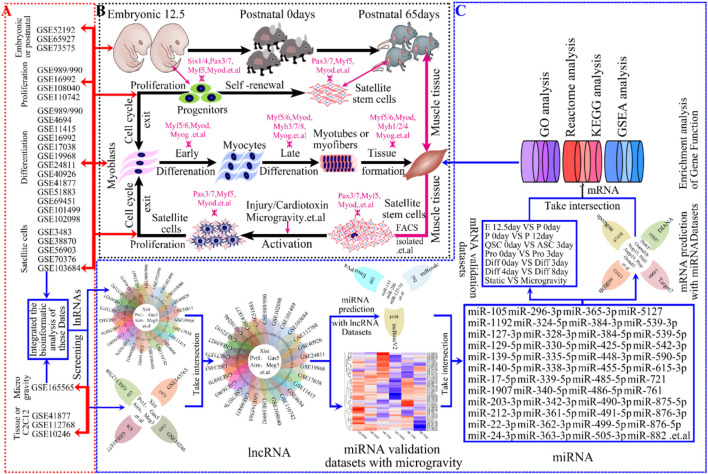
The progression of skeletal muscle development and Schematic workflow of screening lncRNAs and bioinformatics analyses in skeletal muscle. **(A)** Study flow diagram showing GEO datasets used for analyses in skeletal muscle development. **(B)** Hierarchy of progression of skeletal muscle development contained embryonic development, postnatal growth, cell proliferation, cell differentiation, muscle regeneration and so on. For each step, markers for the early and late differentiation are indicated. The genes indicated in magenta encode transcription factors or sarcomeric and associated proteins. **(C)** Schematic illustration of an integrative computational analysis to map, annotate and reconstruct, to screen, validate and gene enrichment analysis lncRNAs in skeletal muscle development, such as KEGG, GO, Reactome, GSEA enrichment analysis and so on.

Skeletal muscle, constituting approximately 40% of body mass in mammals, is essential for posture and movement (Liu et al., 2019a; Chen et al., 2018a) and functions as an endocrine, thermogenic, and metabolic organ (Pant et al., 2016; Egan and Zierath, 2013; Pedersen and Febbraio, 2012; Lee et al., 1985). Muscle development (Zhu et al., 2021), including embryonic and postnatal growth as well as regeneration after injury (Pant et al., 2016; Egan and Zierath, 2013; Pedersen and Febbraio, 2012), plays a crucial for muscle formation and maintenance (Figure 1B), significantly impacting embryonic development. Despite its importance, the molecular mechanisms behind it remain unclear.

Skeletal muscle development spans from mouse embryonic stages to maturity, involving myotome progenitor proliferation, differentiation, and stem cell formation during embryogenesis (Lander, 2011; Plikus et al., 2021; Hemberger et al., 2020; Gupta et al., 2021; Bentzinger et al., 2012; Chal and Pourquie, 2017; Dumont et al., 2015; Hnia et al., 2019) (Lander, 2011; Plikus et al., 2021; Hemberger et al., 2020; Gupta et al., 2021; Bentzinger et al., 2012; Chal and Pourquie, 2017; Dumont et al., 2015; Hnia et al., 2019). Postnatally, muscle cells enhance function through improved endurance, energy metabolism, and cytokine secretion (Bentzinger et al., 2012; Chal and Pourquie, 2017; Dumont et al., 2015; Hnia et al., 2019; Pillon et al., 2020; Zhang et al., 2018a). In contrast, skeletal muscle regeneration (Liu et al., 2017b; Goel et al., 2017) involves the activation, proliferation, and differentiation of quiescent satellite cells, in response to environmental stresses like microgravity. Recent studies show that skeletal muscle development and regeneration are regulated by cell cycle proteins, myogenic transcription factors, cytokines, and noncoding RNAs (Pedersen and Febbraio, 2012; Ciemerych and Sicinski, 2005; Braun and Gautel, 2011; Butchart et al., 2016; Ballarino et al., 2016).

Long noncoding RNAs (LncRNAs), over 200 nucleotides long and non-protein-coding, play roles in epigenetics, gene transcription, and protein expression by sponging miRNAs or interacting with mRNAs (Signal et al., 2016; Ponting et al., 2009; Wang et al., 2021; Yao et al., 2019; Chen, 2016; Kallen et al., 2013; Sui et al., 2019). Accumulating evidence indicates that (Butchart et al., 2016; Li et al., 2018; Goncalves and Armand, 2017; Zhao et al., 2019) lncRNAs influence skeletal muscle development and atrophy by acting as ceRNAs. Examples include LINC00961 (Matsumoto et al., 2017), LncMyoD (Gong et al., 2015), lncRNA Neat1 (Wang et al., 2019a), lncRNA YY1 (Zhou et al., 2015) and lncRNA Malat1 (Han et al., 2015; Chen et al., 2017),. Additionally, lncRNAs like lncRNA MAR1 (Zhang et al., 2018b), Linc-RAM (Yu et al., 2017), lincRNA-MD1 (Legnini et al., 2014), H19 (Kallen et al., 2013), LncRNA-mg (Zhu et al., 2017), and lincRNA Yam-1 (Lu et al., 2013) regulate both muscle proliferation and differentiation. And lncRNAs regulate muscle atrophy by influencing mitochondrial function, cell autophagy, apoptosis, and myofiber size, with examples including lncMUMA (Zhang et al., 2018c), LncIRS1 (Li et al., 2019), lncRNA SMN-AS1 (d'Ydewalle et al., 2017), lncRNA Pvt1 (Alessio et al., 2019). Dysregulated lncRNA expression is linked to skeletal muscle development and related diseases, such as DMD and muscle wasting. Therefore, studying lncRNA functions in muscle development is crucial.

Technological advancements have facilitated the use of GEO Datasets to study lncRNA in skeletal muscle development and environmental responses. Despite over 26 published GEO datasets on mouse muscle development (Figure 1B), comprehensive analysis remains limited. Our global R-analysis of these datasets identified key roles for lncRNAs Xist, Gas5, Pvt1, Airn, and Meg3 in muscle development and microgravity-induced damage (Figure 1). For understanding the mechanisms and signaling pathways involved in skeletal muscle development could shed light on muscle-related diseases and advance personalized treatments.

## 2 Methods and materials

### 2.1 Collection and processing of publicly available GEO datasets

Publicly available GEO microarray datasets (Table 1) were downloaded from NCBI and re-annotated using R and Perl software. These datasets encompassed topics such as embryonic development, postnatal growth, muscle regeneration, cell proliferation, and cell differentiation. Gene symbols for lncRNA and protein-coding mRNA were annotated and extracted using R and Perl from platforms and GRCm39 (https://www.gencodegenes.org/mouse/). Final statistical analyses involved 26 studies from various microarray data (Table 1). We intersected these 26 GEO databases to identify key lncRNAs. Datasets for different phases (e.g., embryonic development, postnatal growth, muscle regeneration) were integrated by time-points (Table 1). The “limma” R package (Ritchie et al., 2015) was used for differential gene expression analysis in R. Results were visualized using heatmaps, volcano plots, bar graphs, and Venn diagrams with “pheatmap” (Metsalu and Vilo, 2015) and “ggplot2” (Ito and Murphy, 2013) R packages. More details are provided in Table 1, and the workflow is shown in Figure 1A.

**TABLE 1:** Datasets used in this study.

GEO	Experiment type	Type or phase	Protocol (time point)	Website	References
GSE52192	Expression profiling	Embryonic and postnatal Phase	Embryonic 12.5day (3 times), Postnatal 1 (8 times), 12 (4 times), 28(3 times), 65day (4 times)	https://www.ncbi.nlm.nih.gov/geo/query/acc.cgi?acc=GSE52192	Mourikis et al. (2012)
GSE65927	Expression profiling			https://www.ncbi.nlm.nih.gov/geo/query/acc.cgi?acc=GSE65927	Kostallari et al. (2015)
GSE73575	NC-RNA profiling			https://www.ncbi.nlm.nih.gov/geo/query/acc.cgi?acc=GSE73575	NR
GSE3483	Expression profiling	Muscle regeneration Phase	Quiescent satellite cells 0day (14 times), Activated satellite cells 12 h (3 times), 2day (7 times), 3day (6 times), 4day (7 times), 8day (3 times)	https://www.ncbi.nlm.nih.gov/geo/query/acc.cgi?acc=GSE3483	Fukada et al. (2007)
GSE38870	Expression profiling			https://www.ncbi.nlm.nih.gov/geo/query/acc.cgi?acc=GSE38870	Farina et al. (2012)
GSE56903	Expression profiling			https://www.ncbi.nlm.nih.gov/geo/query/acc.cgi?acc=GSE56903	Ogawa et al. (2015)
GSE70376	Expression profiling			https://www.ncbi.nlm.nih.gov/geo/query/acc.cgi?acc=GSE70376	Garcia-Prat et al. (2016)
GSE103684	Expression profiling			https://www.ncbi.nlm.nih.gov/geo/query/acc.cgi?acc=GSE103684	Latroche et al. (2017)
GSE989	Expression profiling	Proliferation Phase	Proliferation 0day (12 times), 1day (6 times), 2day (3 times), 3day (6 times)	https://www.ncbi.nlm.nih.gov/geo/query/acc.cgi?acc=GSE989	Tomczak et al. (2004)
GSE990	Expression profiling			https://www.ncbi.nlm.nih.gov/geo/query/acc.cgi?acc=GSE990	Tomczak et al. (2004)
GSE16992	Expression profiling			https://www.ncbi.nlm.nih.gov/geo/query/acc.cgi?acc=GSE16992	Rajan et al. (2012)
GSE108040	Expression profiling			https://www.ncbi.nlm.nih.gov/geo/query/acc.cgi?acc=GSE108040	Wust et al. (2018)
GSE110742	Expression profiling			https://www.ncbi.nlm.nih.gov/geo/query/acc.cgi?acc=GSE110742	NR
GSE989	Expression profiling	Differentiation Phase	Differentiation 0day (41 times), 1 h (6 times), 2 h (6 times), 3 h (6 times), 6 h (9 times), 9 h (6 times), 12 h (6 times), 1day (14 times), 2day (20 times), 3day (9 times), 4day (18 times), 5day (12 times), 6day (18 times), 8day (3 times), 10day (3 times)	https://www.ncbi.nlm.nih.gov/geo/query/acc.cgi?acc=GSE989	Tomczak et al. (2004)
GSE990	Expression profiling			https://www.ncbi.nlm.nih.gov/geo/query/acc.cgi?acc=GSE990	Tomczak et al. (2004)
GSE4694	Expression profiling			https://www.ncbi.nlm.nih.gov/geo/query/acc.cgi?acc=GSE4694	Chen et al. (2006)
GSE11415	Expression profiling			https://www.ncbi.nlm.nih.gov/geo/query/acc.cgi?acc=GSE11415	Ma et al. (2008)
GSE16992	Expression profiling			https://www.ncbi.nlm.nih.gov/geo/query/acc.cgi?acc=GSE16992	Rajan et al. (2012)
GSE17038	Expression profiling			https://www.ncbi.nlm.nih.gov/geo/query/acc.cgi?acc=GSE17038	Rajan et al. (2012)
GSE19968	Expression profiling			https://www.ncbi.nlm.nih.gov/geo/query/acc.cgi?acc=GSE19968	Liu et al. (2010)
GSE24811	Expression profiling			https://www.ncbi.nlm.nih.gov/geo/query/acc.cgi?acc=GSE24811	Soleimani et al. (2012)
GSE40926	Expression profiling			https://www.ncbi.nlm.nih.gov/geo/query/acc.cgi?acc=GSE40926	Bricchi et al. (2012)
GSE41877	Expression profiling			https://www.ncbi.nlm.nih.gov/geo/query/acc.cgi?acc=GSE41877	Frangini et al. (2013)
GSE51883	Expression profiling			https://www.ncbi.nlm.nih.gov/geo/query/acc.cgi?acc=GSE51883	Hupkes et al. (2014)
GSE69451	Expression profiling			https://www.ncbi.nlm.nih.gov/geo/query/acc.cgi?acc=GSE69451	Militello et al. (2018)
GSE101499	NC-RNA profiling			https://www.ncbi.nlm.nih.gov/geo/query/acc.cgi?acc=GSE101499	Chen et al. (2018b)
GSE102098	NC-RNA profiling			https://www.ncbi.nlm.nih.gov/geo/query/acc.cgi?acc=GSE102098	Jin et al. (2018)
GSE41877	Expression profiling	Muscle tissue and C2C12 expression data	C2C12 (3 times), EDL_Muscle (3 times), Soleus_Muscle (3 times)	https://www.ncbi.nlm.nih.gov/geo/query/acc.cgi?acc=GSE41877	Frangini et al. (2013)
GSE112768	Expression profiling		EDL-Cyt (3 times), EDL-Nuc (3 times), SOL-Cyt (3 times), SOL-Nuc (3 times)	https://www.ncbi.nlm.nih.gov/geo/query/acc.cgi?acc=GSE112768	Alessio et al. (2019)
GSE10246	Expression profiling		C2C12 (2 times), Muscle tissue (2 times)	https://www.ncbi.nlm.nih.gov/geo/query/acc.cgi?acc=GSE10246	Lattin et al. (2008)
GSE165565	Expression profiling	Muscle wasting (atrophy) Phase	Static (3 times), Microgravity (3 times)	https://www.ncbi.nlm.nih.gov/geo/query/acc.cgi?acc=GSE165565	Genchi et al. (2021)

NC-RNA, profiling: Non-coding RNA, profiling; GSE989/990: GSE989 and GSE990; EDL: extensor digitorum longus; SOL: soleus; Cyt: Cytoplasmic; NR: not reference.

### 2.2 Prediction of targeting relationship and construction of the lncRNA-miRNA-mRNA ceRNA regulatory network

To investigate post-transcriptional regulation in skeletal muscle development, we constructed a lncRNA-miRNA-mRNA ceRNA network. The workflow included predicting lncRNA-miRNA interactions using the miRcode (Jeggari et al., 2012), lncBaseV2 (Karagkouni et al., 2020), and Enco/PV4 (Teng et al., 2020; Li et al., 2014) (Integration database with ENCORI and NPInterv4) databases, followed by intersecting these predictions. Predicted miRNAs were filtered based on expression datasets from hindlimb suspension simulated microgravity. Subsequently, miRNA-mRNA interactions were identified through intersecting results from miRcode (Jeggari et al., 2012), DIANA (Karagkouni et al., 2018), miRDB (Chen and Wang, 2020), and TarBase7 (Agarwal et al., 2015) databases. Finally, a ceRNA regulatory network comprising 5 lncRNAs, 36 miRNAs, and 108 mRNAs was built using R software, with visualization facilitated by the “ggalluvial” R package (Rosvall and Bergstrom, 2010; Liu et al., 2017a). This comprehensive approach allowed us to elucidate the intricate regulatory relationships within the network.

### 2.3 Enrichment analysis

To investigate the functional ceRNA regulatory network of lncRNA-miRNA-mRNA, we performed an enrichment analysis on predicted mRNAs. This involves identifying mRNAs by intersecting predicted mRNAs with various stages of skeletal muscle development, including comparisons between embryonic and postnatal phases, quiescent and activated satellite cells, and different differentiation stages. Secondly, we applied GO (Lei et al., 2019; Sun et al., 2019) (Gene Ontology) analysis to the primary screened mRNAs for enrichment of biological processes, cellular components, and molecular functions. The Reactome (Jassal et al., 2020) knowledge base was used to further validate their molecular functions. KEGG (Lei et al., 2019; Sun et al., 2019) analysis identified potential mRNA pathways, while GSEA (Cheng et al., 2021) assessed gene distribution trends to determine their phenotypic contributions. GO and KEGG enrichment were conducted with the “clusterProfiler” R package (Wu et al., 2021), while Reactome and GSEA analyses were conducted using the “ReactomePA” (Yu and He, 2016) and “GSEA” (Reimand et al., 2019) R packages, respectively. Visualization of the GO, KEGG, Reactome, and GSEA analysis results was achieved using the “ggplot2″ R package.

### 2.4 Statistical analyses

Data is presented as mean ± SEM. All statistical analyses were conducted with R software, version 4.0.5 (R Foundation for Statistical Computing, Vienna, Austria). The R software ggpubr, reshape2, rstatix package et al. was used to perform statistical analyses. Analysis results were plotted using the R packages ggplot2. Student’s t-test was utilized to compare the differences between the two groups. Comparisons were considered statistically significant at *p* ≤ 0.05.

### 2.5 Data availability

Further information on research design is available in the GEO datasets linked to this article (Table 1). All data were freely accessible online.

## 3 Results

### 3.1 Screening of candidate lncRNAs from public GEO datasets

To identify lncRNAs involved in skeletal muscle development, we re-annotated and analyzed 26 publicly available GEO datasets using R and Perl software (Figures 1A, C; Table 1). These datasets, sourced from NCBI, encompass embryonic and postnatal stages (3 datasets), muscle regeneration (5 datasets), proliferation (5 datasets), differentiation (14 datasets), muscle tissue or C2C12 expression (3 datasets), and muscle wasting expression (1 datasets). Our study workflow is depicted in Figure 1C, with detailed information provided in Table 1. Through the analysis of these GEO databases (Table 2), We identified the lncRNAs—Xist, Gas, Pvt1, Airn, and Meg3 (Figure 1C; Table 1) — as having regulatory potential in skeletal muscle development (Figure 1B). These specific lncRNAs were selected based on their differential expression patterns observed across various stages and conditions of muscle development and function.

**TABLE 2:** Screening of five candidate lncRNAs from 26 public datasets.

Gene	GSE101499	GSE102098	GSE10246	GSE103684	GSE108040	GSE110742	GSE112768	GSE11415	GSE165565	GSE16992	GSE17038	GSE19968	GSE24811
Airn	1	1	1	1	1	1	1	1	1	1	1	1	1
Pvt1	1	1	1	1	1	1	1	1	1	1	1	1	1
Gas5	1	1	1	1	1	1	1	1	1	1	1	1	1
Meg3	1	1	1	1	1	1	1	1	1	1	1	1	1
Xist	1	1	1	1	1	1	1	1	1	1	1	1	1
Dleu2	1	1	1	1	1	1	1	1	1	1	1	1	1
R74862	1	0	1	1	1	1	1	1	1	1	1	1	1
U90926	1	0	1	1	1	1	0	1	1	1	1	1	1
Malat1	1	1	1	1	1	1	1	1	1	1	1	1	1
Snhg5	1	0	1	1	1	1	0	1	1	1	1	1	1

GSE3483	GSE38870	GSE40926	GSE41877	GSE4694	GSE51883	GSE52192	GSE56903	GSE65927	GSE69451	GSE70376	GSE73575	GSE989/990	Sum
1	1	1	1	1	1	1	1	1	1	1	1	1	26
1	1	1	1	1	1	1	1	1	1	1	1	1	26
1	1	1	1	1	1	1	1	1	1	1	1	1	26
1	1	1	1	1	1	1	1	1	1	1	1	1	26
1	1	1	1	1	1	1	1	1	1	1	1	1	26
1	1	1	1	1	1	1	1	1	1	1	1	0	25
1	1	1	1	1	1	1	1	1	1	1	1	1	25
1	1	1	1	1	1	1	1	1	1	1	1	1	24
1	1	0	1	1	1	1	1	1	1	1	1	0	24
1	1	1	1	1	1	1	1	1	1	1	1	1	24

GSE989/990: GSE989 and GSE990; “1” represents that this lncRNA, is present in the datasets; “0” represents that this lncRNA, is not present in the datasets; only top 10 lmcRNA, were shown in the Table.

**FIGURE 2:**
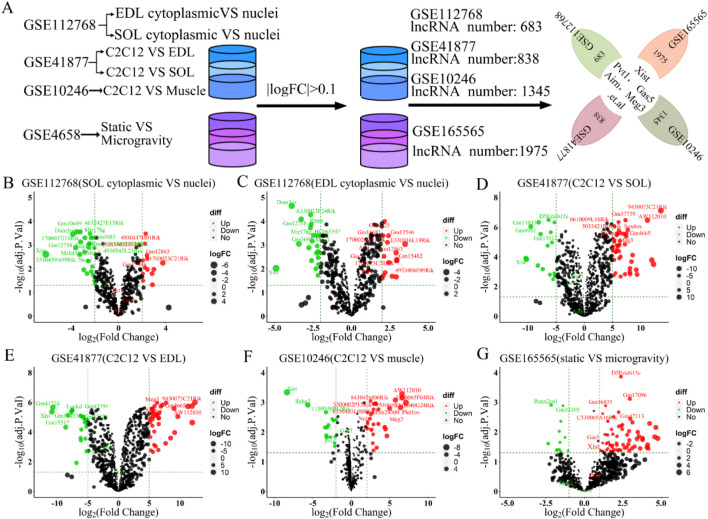
The 5 candidate lncRNAs (lncRNA Xist, lncRNA Gas5, lncRNA Pvt1, lncRNA Airn and lncRNA Meg3) were aberrantly expressed in GSE112768, GSE41877, GSE10246, and GSE165565 datasets. **(A)** Schematic workflow for statistical analysis process. A volcano plot was used to show the differentially expressed lncRNAs. The negative Log2-adjusted *p*-values (y-axis) are plotted against the Log2 fold changes in expression (x-axis). The horizontal dashed line indicates the threshold for significance (*p* ≤ 0.05) and the vertical dashed line indicates the upregulated (right side) and downregulated (left side) lncRNAs. **(B)** Volcano plot showing the differentially expressed lncRNAs between soleus myofibers nucleus *versus* cytoplasm in GSE112768. **(C)** Volcano plot showing the differentially expressed lncRNAs between EDL myofibers nucleus *versus* cytoplasm in GSE112768. **(D)** Volcano plot presenting the differentially expressed lncRNAs between C2C12 *versus* soleus in GSE41877. **(E)** Volcano plot presenting the differentially expressed lncRNAs between C2C12 *versus* EDL in GSE41877. **(F)** Volcano plot illustrating the differentially expressed lncRNAs between C2C12 *versus* muscle in GSE10246. **(G)** Volcano plot displaying the differentially expressed lncRNAs between static vs. simulated microgravity in GSE165565. EDL: extensor digitorum longus, SOL: Soleus.

Muscle atrophy in humans and mammals exposed to microgravity leads to muscle function loss or pathological changes (Vico and Hargens, 2018; Garrett-Bakelman et al., 2019). To compare lncRNA expression between normal and atrophied muscle, we re-annotated and analyzed GEO datasets from three studies on normal skeletal muscle (GSE112768, GSE877, GSE10246) and one on microgravity-induced muscle (GSE165565) using R and Perl software (Figure 1C). By setting the logFC threshold to greater than 0.1, we identified 683, 838, 1,345, and 1975 lncRNAs retained in GSE112768, GSE877, GSE10246, and GSE165565, respectively (Figure 2A). We then re-screened and validated lncRNAs Xist, Gas5, Pvt1, Airn, and Meg3 across all four datasets (Figure 2A). In muscle tissue (SOL or EDL), lncRNA Xist and Meg3 showed significant downregulation, while lncRNAs Gas5, Pvt1, and Airn were upregulated (Figures 2B, C). In C2C12 cells *versus* muscle tissue (SOL or EDL), lncRNAs Xist, Gas5, Pvt1, and Airn were significantly downregulated, with Xist decreasing over 5-fold, while Meg3 was upregulated about 5-fold (Figures 2D, E). In GSE10246, Xist, Gas5, and Pvt1 were downregulated, while Meg3 and Airn were upregulated (Figure 2F). In GSE165565, Xist expression increased 1.7 times in microgravity compared to static conditions, while Meg3, Gas5, Pvt1, and Airn showed no significant change (Figure 2G). These results indicate that the lncRNAs Xist, Gas5, Pvt1, Airn, and Meg3 exhibit differential expression and diverse functions across various contexts, potentially regulating skeletal muscle development. Notably, Xist stands out for its significant changes across different conditions, suggesting a crucial role in skeletal muscle regulation.

### 3.2 The role of lncRNAs in skeletal muscle development: A focus on Xist and its regulatory significance

Understanding skeletal muscle development (Zhu et al., 2021), which includes embryonic formation, postnatal growth, and injury recovery, is essential for maintaining physiological functions (Hemberger et al., 2020; Gupta et al., 2021; Chal and Pourquie, 2017; Hnia et al., 2019). This process is driven by myogenesis, involving muscle cell proliferation, differentiation, and fusion (Bentzinger et al., 2012; Dumont et al., 2015; Liu et al., 2017a; Goel et al., 2017). LncRNAs (Gupta et al., 2021; Ciemerych and Sicinski, 2005; Spittau et al., 2020; Chini and Hanganu-Opatz, 2021) significantly influence skeletal muscle development during embryonic and postnatal growth in mice, contributing to muscle maturation (Figure 1B). By analyzing 23 GEO datasets (Figures 1A, C), we explore the molecular roles of specific lncRNAs—Xist, Gas5, Pvt1, Airn, and Meg3—at different developmental stage (Figure 1; Table1).

**FIGURE 3:**
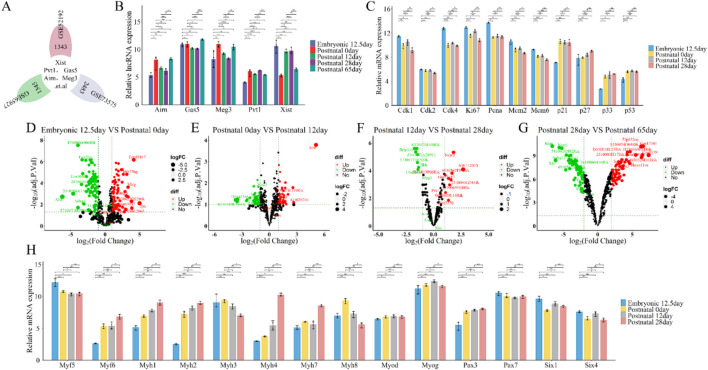
Functional analysis of differentially expressed lncRNAs (lncRNA Xist, lncRNA Pvt1, lncRNA Gas5, lncRNA Airn and lncRNA Meg3) in stages of embryonic development and postnatal growth. **(A)** Flower plot diagram presents the overlap of common lncRNAs in GSE52192, GSE73575 and GSE65927 datasets. **(B)** The expression of lncRNA Xist, lncRNA Pvt1, lncRNA Gas5, lncRNA Airn and lncRNA Meg3 during Embryonic 12.5 days, Postnatal 0day, Postnatal 12day, Postnatal 28 days, Postnatal 65 days. **(C)** The expression of cell cycle related protein and inhibitor at Embryonic 12.5 days, Postnatal 0 day, Postnatal 12 days, Postnatal 28 days **(D)** Volcano plot showing the differentially expressed lncRNAs between Embryonic 12.5 days *versus* Postnatal 0 day. **(E)** Volcano plot presenting the differentially expressed lncRNAs between Postnatal 0day *versus* Postnatal 12days **(F)** Volcano plot illustrating the differentially expressed lncRNAs between Postnatal 12 days *versus* Postnatal 28 days. **(G)** Volcano plot displaying the differentially expressed lncRNAs between Postnatal 28 days *versus* Postnatal 65 days **(H)** The expression of key myogenic regulators during Embryonic 12.5 days, Postnatal 0 days, Postnatal 12 days, Postnatal 28 days. A volcano plot was used to show the differentially expressed lncRNAs. The negative Log2-adjusted P-values (y-axis) are plotted against the Log2 fold changes in expression (x-axis). The horizontal dashed line indicates the threshold for significance (P ≤ 0.05) and the vertical dashed line indicates the upregulated (right side) and downregulated (left side) lncRNAs. The data are presented as the means ± S.D. of the samples from all different samples. The p-values were calculated using Student’s t-test. NS, non-significant; *p < 0.05; **p < 0.01; ***p < 0.0001. **(E)** Embryonic; P: Postnatal.

To examine the roles and expression of these lncRNAs (Figure 3A), we re-annotated and analyzed GEO datasets GSE52192, GSE73575, and GSE65927 using R and Perl software (Figure 1C; Figure 3A). Our results showed that the expression of these lncRNAs’ increased from embryonic day 12.5 to postnatal day 0, decreased until postnatal day 28, and then rose again by postnatal day 65, following a similar pattern for all five lncRNAs (Figure 3B). During embryonic development and postnatal growth, lncRNAs are regulated to ensure proper cell proliferation, differentiation, and apoptosis. For instance, levels of cyclins (Cdk1, Cdk2, Cdk4, Ki67, Pcna, Mcm2, Mcm6) decreased and inhibitors (p21, p27, p33, p53) increased over time (Figure 3C), highlighting their roles in growth. Specific myogenic markers, including Six1/4, Pax3/7, Myf5, Myod, Myog, Myf6, and Myh1/2/3/4/7/8, define each stage of skeletal muscle development. Analysis revealed that Pax3, Myod, Myog, Myf6, and Myh1/2/4/7 levels rise during development, while Myf5, Myh3/8, Pax7, and Six1/4 levels decline (Figure 3H), indicating muscle cell proliferation and differentiation during embryonic and postnatal growth. Volcano plot results further highlighted significant changes in lncRNA Xist expression: it significantly decreased from embryonic day 12.5 to postnatal day 0 (logFC = −6.22, P≤ 0.001, Figure 3D), increased from postnatal day 0 to day 12 (logFC = 5.08, P≤ 0.001, Figure 3E), remained stable from day 12 to day 28 (logFC = −0.01, P≤ 0.98, Figure 3F), and significantly decreased again from day 28 to day 65 (logFC = −6.12, P≤ 0.001, Figure 3G). In contrast, lncRNAs (Pvt1, Gas5, Airn, and Meg3) showed no significant changes (|logFC|≥3, Pvalue≤0.5) during both embryonic development and postnatal growth (Figures 3D–G). These findings suggest that while lncRNAs Pvt1, Gas5, Airn, and Meg3 may play a role in regulating embryonic development and postnatal growth, lncRNA Xist is particularly significant in this regulatory process.

Long non-coding RNAs (lncRNAs) play a pivotal role in muscle injury and are instrumental in skeletal muscle regeneration, a process heavily reliant on satellite cells located beneath the myofiber’s basement membrane (Peault et al., 2007; Tumpel and Rudolph, 2019; Cho et al., 2019). These quiescent progenitors are essential for muscle maintenance, growth, repair, and regeneration. Upon activation, satellite cells (Peault et al., 2007; Tumpel and Rudolph, 2019; Cho et al., 2019; Sirabella et al., 2013) proliferate, differentiate into myoblasts, and fuse to form myotubes. Muscle recovery typically takes 30–90 days, with fundamental repair occurring within 14–30 days. Cell proliferation peaks at 3–4 days, while cell differentiation peaks at 7–10 days (Dumont et al., 2015; Ogawa et al., 2015; Aguilar et al., 2016; Lukjanenko et al., 2013). This timeline remains consistent regardless of the nature or cause of the damage, including that induced by microgravity (Burzyn et al., 2013).

**FIGURE 4:**
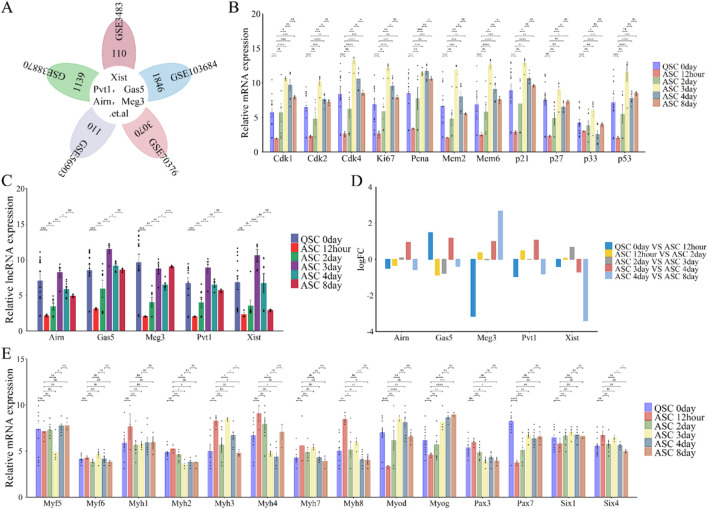
Functional analysis of differentially expressed lncRNAs (lncRNA Xist, lncRNA Pvt1, lncRNA Gas5, lncRNA Airn and lncRNA Meg3) in phase of skeletal muscle regeneration. **(A)** Flower plot diagram shows the overlap of lncRNAs in GSE3483, GSE38870, GSE56903, GSE70376 and GSE103684 datasets. **(B)** The expression of cell cycle related protein and inhibitor at skeletal muscle regeneration. **(C)** The expression of lncRNA Xist, lncRNA Pvt1, lncRNA Gas5, lncRNA Airn and lncRNA Meg3 during E skeletal muscle regeneration. **(D)** Bar graphs presenting the differentially expressed lncRNAs (lncRNA Xist, lncRNA Pvt1, lncRNA Gas5, lncRNA Airn and lncRNA Meg3) in skeletal muscle development. Bar graphs was used to show the differentially expressed lncRNAs. The y-axis is Log2 fold changes in expression and x-axis is lncRNAs. **(E)** The expression of key myogenic regulators during skeletal muscle regeneration. The data are presented as the means ± S.D. of the samples from all different samples. The p-values were calculated using Student’s t-test. NS, non-significant; *p < 0.05; **p < 0.01; ***p < 0.0001. QSC, Quiescent satellite cells; ASC, Activated satellite cells.

We study the roles and expression patterns of lncRNAs Xist, Pvt1, Gas5, Airn, and Meg3 in skeletal muscle regeneration, we re-annotated and integrated data from GEO datasets GSE3483, GSE38870, GSE56903, GSE70376, and GSE103684 using R and Perl (Figures 1C; Figures 4A). Our analysis showed regular changes in lncRNA expression during muscle repair (Figure 4C). Specifically, lncRNA expression decreased from QSC 0 day to ASC 12 h, then increased from ASC 12 h to ASC 3 days, followed by a gradual decline until ASC 8 days, returning to baseline levels (Figures 4C, D). Notably, lncRNA Meg3 surged (logFC≥3) at ASC 8 days, while lncRNA Xist dropped significantly (logFC ≤ −3) at the same time. Other lncRNAs followed a similar trend (Figures 4C, D).

Cyclins, cell cycle inhibitors, and myogenic markers are crucial for muscle regeneration. Cyclin levels (Cdk1, Cdk2, Cdk4, Ki67, Pcna, Mcm2, and Mcm6)increased significantly from 12 h to 3 days after satellite cell activation (ASC), then gradually declined until 8 days (Figure 4B). Similarly, cell cycle checkpoint proteins p21 and p33 rose sharply from 12 h to 3 days before decreasing by 8 days (Figure 4B). In contrast, p27 and p53 showed a steady increase from 12 h to 8 days, except at the 3-day mark (Figure 4B). Myogenic markers indicated that Pax3, Six4, and Myh2/4/7/8 levels declined during regeneration (Figure 4E), while Pax7, Six1, and Myog increased. Myod and Myh3 levels rose significantly from 12 h to 3 days post-ASC, then decreased from 3 to 8 days (Figure 4E). Myf5/6 and Myh1 expression was absent during regeneration (Figure 4E). These findings suggest that lncRNAs, including Xist, Pvt1, Gas5, Airn, and Meg3, regulate skeletal muscle regeneration. Notably, lncRNA Xist plays a particularly significant role.

**FIGURE 5:**
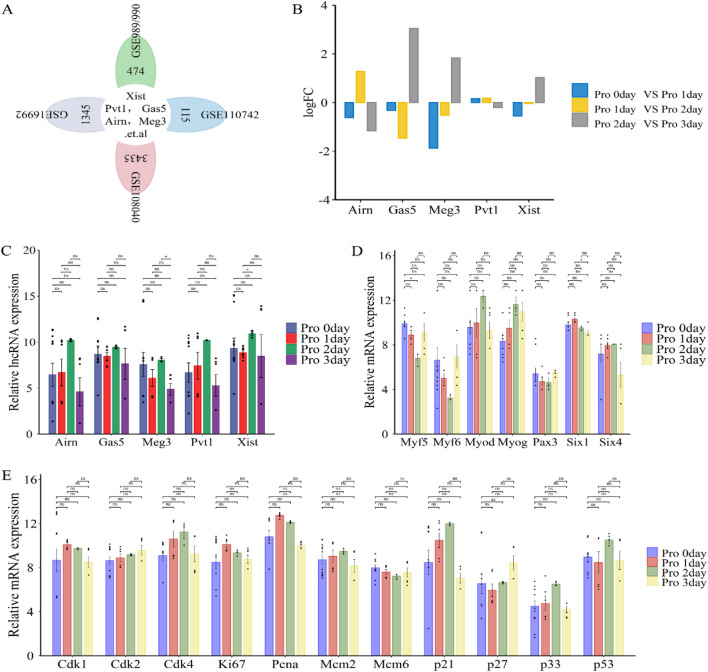
Functional analysis of differentially expressed lncRNAs (lncRNA Xist, lncRNA Pvt1, lncRNA Gas5, lncRNA Airn and lncRNA Meg3) in phase of cell proliferation. **(A)** Flower plot diagram displays the overlap of lncRNAs in GSE989/990, GSE110742, GSE108040 and GSE16992 datasets. **(B)** Bar graphs presenting the differentially expressed lncRNAs (lncRNA Xist, lncRNA Pvt1, lncRNA Gas5, lncRNA Airn and lncRNA Meg3) in cell proliferation. Bar graphs was used to illustrate the differentially expressed lncRNAs. The y-axis is Log2 fold changes in expression and x-axis is lncRNAs. **(C)** The expression of lncRNA Xist, lncRNA Pvt1, lncRNA Gas5, lncRNA Airn and lncRNA Meg3 during cell proliferation. **(D)** The expression of key myogenic regulators during cell proliferation. **(E)** The expression of cell cycle related protein and inhibitor during cell proliferation. The data are presented as the means ± S.D. of the samples from all different samples. The p-values were calculated using Student’s t-test. NS, non-significant; *p < 0.05; **p < 0.01; ***p < 0.0001. Pro, proliferation; GSE989/990, GSE989 and GSE990.

LncRNAs such as Xist, Pvt1, Gas5, Airn, and Meg3 (Figure 5A) play a crucial role in regulating cell proliferation during skeletal muscle development and regeneration (Zhao et al., 2019; Luo et al., 2021). This process is essential for muscle growth and repair and precedes cell differentiation. To investigate their roles, we re-annotated and integrated analysis of GEO datasets (GSE989/990, GSE110742, GSE108040, and GSE16992) using R and Perl software (Figures 1C; Figures 5A).

Our analysis revealed that lncRNAs like Airn and Pvt1 were significantly upregulated from day 0 to day 2 of proliferation, then downregulated from day 2 to day 3 (Figures 5B, C). Conversely, lncRNAs such as Gas5, Meg3, and Xist showed a decrease from day 0 to day 1, followed by a progressive increase from day 1 to day 2, and were upregulated again from day 2 to day 3 (Figures 5B, C). These fluctuations align with the regulation of cell cycle progression by various proteins, including cyclins, CDKs, and inhibitors like CDK1, CDK2, CDK4, Mcm2, Mcm6, P21, P27, P57, Ki67, and PCNA (Ponnusamy et al., 2017; Mahdessian et al., 2021; Huang et al., 2017). Specifically, Cdk2 and p27 levels increased during the proliferation cycle, while Mcm6 expression notably decreased from day 0 to day 3 of proliferation (Figure 5E). Additionally, Cdk1, Ki67, and Pcna expression peaked at proliferation day 1 and decrease by day 3, while Cdk4, Mcm2, p21, p33, and p53 increased from day 0 to day 2 and then decline by day 3 (Figure 5E). These patterns suggest that muscle cells begin differentiating around day 3. During skeletal muscle cell proliferation, markers such as Six1/4, Pax3, Myf5, Myf6, Myod, and Myog are expressed (Figure 5D). Our analysis showed that Myod, Myog, and Six4 levels increased from day 0 to day 2, and decreased from day 2 to day 3. Six1 peaked at day 1 and declined thereafter. Pax3 and Myf5/6 decreased from day 0 to day 2 and increased from day 2 to day 3 (Figure 5D). This confirmed the transition from proliferation to differentiation. Overall, our findings indicate that lncRNAs, including Xist, Pvt1, Gas5, Airn, and Meg3, may play critical roles in promoting cell proliferation during muscle development or regeneration.

**FIGURE 6:**
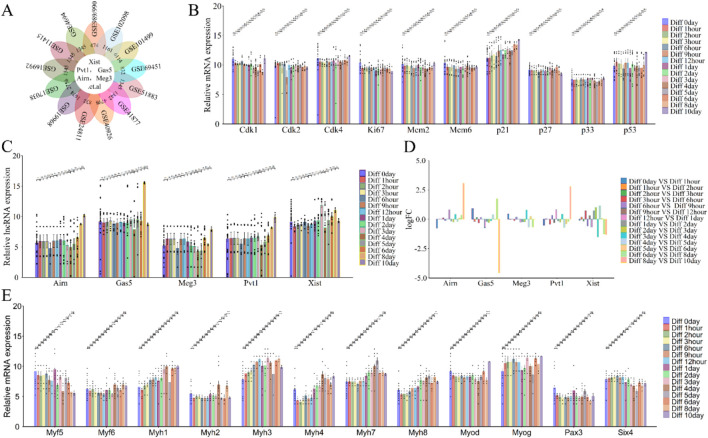
Functional analysis of differentially expressed lncRNAs (lncRNA Xist, lncRNA Pvt1, lncRNA Gas5, lncRNA Airn and lncRNA Meg3) in phase of cell differentiation. **(A)** Flower plot diagram illustrates the intersection of the 13 datasets analyzed (GSE989/990, GSE102098, GSE101499 and so on). **(B)** The expression of cell cycle related protein and inhibitor during cell differentiation. **(C)** The expression of lncRNA Xist, lncRNA Pvt1, lncRNA Gas5, lncRNA Airn and lncRNA Meg3 during cell differentiation. **(D)** Bar graphs showing the differentially expressed lncRNAs (lncRNA Xist, lncRNA Pvt1, lncRNA Gas5, lncRNA Airn and lncRNA Meg3) in cell differentiation. Bar graphs was used to illustrate the differentially expressed lncRNAs. The y-axis is Log2 fold changes in expression and x-axis is lncRNAs. **(E)** The expression of key myogenic regulators during cell differentiation. The data are presented as the means ± S.D. of the samples from all different samples. The p-values were calculated using Student’s t-test. NS, non-significant; *p < 0.05; **p < 0.01; ***p < 0.0001. Diff, Differentiation; GSE989/990, GSE989 and GSE990.

Long non-coding RNAs (lncRNAs) such as Xist, Pvt1, Gas5, Airn, and Meg3 play crucial roles in regulating cell differentiation, which is essential for skeletal muscle development and regeneration (Bentzinger et al., 2012; Hnia et al., 2019; Tomczak et al., 2004; Rajan et al., 2012)**.** To investigate the function and expression of lncRNAs during this process, we re-annotated and analyzed GEO datasets (e.g., GSE989/990, GSE102098, GSE101499) using R and Perl (Figure 1C; Figure 6A). The analysis revealed varying changes in lncRNAs expression throughout cell differentiation (Figures 6C, D).

From day 0 to day 3 of differentiation, lncRNA Airn and Meg3 expressions decreased, while lncRNA Xist increased; lncRNA Pvt1 and Gas5 showed minimal changes (Figures 6C, D). From day 4 to day 10, lncRNA Xist, Pvt1, Airn, and Meg3 continued to rise, although the increase in Gas5 was less pronounced (Figures 6C, D). Cell differentiation (Ponnusamy et al., 2017; Mahdessian et al., 2021; Huang et al., 2017) is closely linked to cell-cycle exit and is regulated by factors such as cyclins, CDKs, and proliferation markers (CDK1, CDK2, CDK4, Mcm2, Mcm6, P21, P27, P57, Ki67, and Pcna). Our analysis showed that the expression of Cdk1, Ki67, Mcm2, Mcm6, Cdk2, Cdk4, p27, p33, and p53 remained stable during differentiation, while p21 exhibited a consistent upward trend (Figure 6B). Cyclin and differentiation marker analysis confirmed muscle cell differentiation with increased Myf6, Myog, and Myh1/3/4/7/8 levels, and decreased levels of Myf5 and Six4. Myod, Pax3, and Myh2 showed fewer clear trends (Figure 6E). These results indicate that lncRNAs Xist, Pvt1, Gas5, Airn, and Meg3 play roles in muscle cell differentiation, supporting our hypothesis on skeletal muscle development or regeneration.

### 3.3 LncRNA–miRNA–mRNA ceRNA network construction and visualization

**FIGURE 7:**
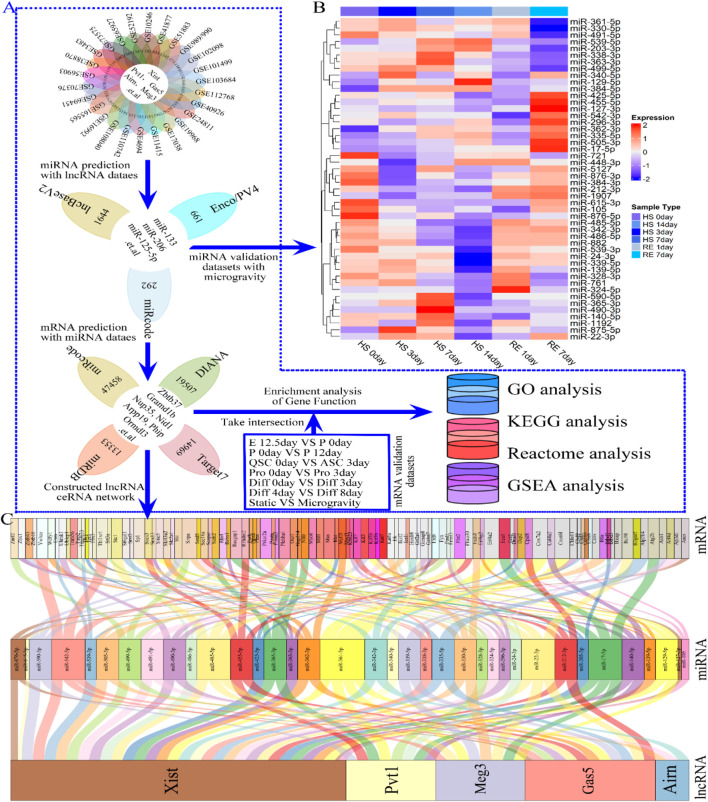
Flowchart indicating the downstream analysis of the lncRNAs (lncRNA Xist, lncRNA Pvt1, lncRNA Gas5, lncRNA Airn and lncRNA Meg3) and Verification of miRNA differential expression. **(A)** Flow chart of the ceRNA network construction. Three independent lncRNA target databases (miRcode, lncBaseV2 and Enco/PV4) were used to predict the potential miRNAs, and screening for miRNAs highly related to microgravity-induced muscle atrophy or regeneration. Subsequently, four independent miRNAs target databases (miRcode, DIANA, miRDB and Target7) were used to predict the potential mRNAs, and using to constructing lncRNA–miRNA–mRNA ceRNA regulatory network. **(B)** The heat map showing the differentially expressed miRNA during HS 0day, HS 3 dayS, HS 7 dayS HS 14 dayS, RE 1day and RE 14days **(C)** Alluvial diagram demonstrates the relationships of lncRNA-miRNA-mRNA ceRNA regulatory network. The interaction network was constructed with there were 5 lncRNAs, 36 miRNAs, and approximately 100 mRNAs. Enco/PV4, ENCORI and NPInterv4; Target7, Targetscan7; GSE989/990, GSE989 and GSE990; HS, hindlimb suspension; RE, Recovery exercise.

Our research suggests that lncRNAs Xist, Pvt1, Gas5, Airn, and Meg3 may influence various stages of skeletal muscle development, including cell proliferation and differentiation. To explore their roles, we constructed a ceRNA network based on predicted lncRNA-miRNA and miRNA-mRNA interactions from relevant datasets (Figure 7A). We identified 108 miRNAs by intersecting data from miRcode, lncBaseV2, and Enco/PV4 (Figure 7A; Supplementary Table S1). These miRNAs were validated using expression datasets from hindlimb suspension (HS) simulating microgravity. The results showed that miRNA levels, including miR-212-3p, miR-485-3p, miR-342-3p, miR-539-3p, miR-486-5p, and miR-24-3p, decreased gradually from HS 0 to HS 14 days and then quickly returned to baseline during recovery exercise (RE) from RE 1 to RE 7 days. Conversely, miRNAs such as miR-203-3p, miR-539-5p, miR-129-5p, miR-361-5p, and miR-489-5p exhibited opposite trends during HS and RE (Figure 7B; Supplementary Figure S1). These findings suggest that the predicted miRNAs have regulatory potential in skeletal muscle development.

By analyzing four datasets (miRcode, DIANA, miRDB, and Target7), we identified almost 12,000 mRNAs (Figure 7A; Supplementary Table S1). We constructed a ceRNA regulatory network with 5 lncRNAs, 36 miRNAs, and 108 mRNAs (Figure 7C), highlighting additional targeting relationships in Supplementary Table S1. Notably, lncRNA Xist (Figure 7C) negatively regulates most miRNAs to boost downstream mRNA expression, influencing skeletal muscle development (e.g., lncRNA Xist/miR-485-5p/Msn and lncRNA Xist/miR-342-3p/Hoxb8). Similar interactions were observed for lncRNAs Airn, Gas5, Meg3, and Pvt1, with specific interactions such as lncRNA Pvt1/miR-455-5p/Fbxo30 and lncRNA Meg3/miR-330-5p/Fads1 (Figure 7C). To investigate how the ceRNA network regulates downstream biological functions and signaling pathways, we conducted GO, Reactome, KEGG, and GSAEA enrichment analyses on the intersection of 12,000 predicted mRNAs and those from each phase of skeletal muscle development using R software (Figure 1C; Figure 7A). Gene network analysis proved to be a valuable tool for studying lncRNA functional changes and understanding selective activation of signaling pathways in skeletal muscle development and regeneration.

### 3.4 Gene functional enrichment analysis during skeletal muscle development and regeneration

**FIGURE 8:**
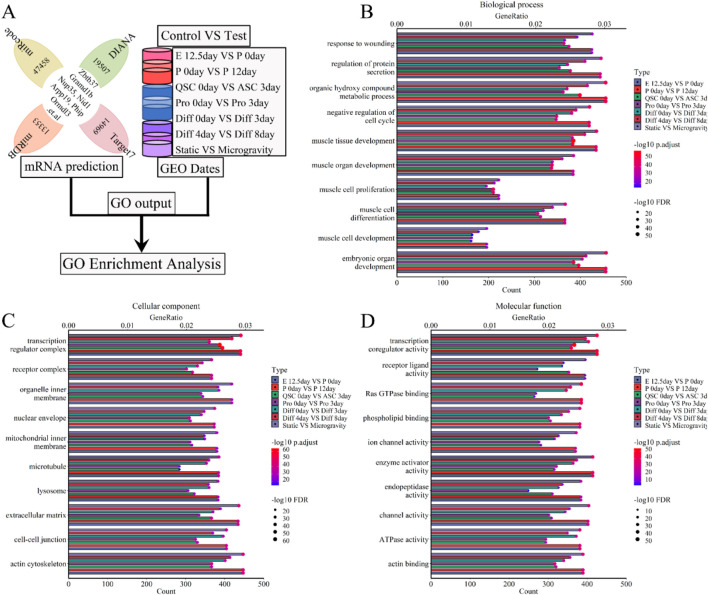
GO enrichment analysis for the lncRNA-miRNA-mRNA ceRNA regulatory network of various stages of skeletal muscle development. **(A)** The workflow showed that the mRNA expression of various stages of skeletal muscle development were enriched with GO enrichment analysis. **(B)** The bubble pattern and bar chart show the 10 randomly chosen biological process with GeneRatio and gene count. The skeletal muscle development, wounding respond and protein secretion et al. correlated with gene enrichment. **(C)** The bubble pattern and bar chart display the 10 randomly chosen cellular component with GeneRatio and gene count. The cell membrane, microtubule, lysosome and mitochondrial et al. associated with gene enrichment. **(D)** The bubble pattern and bar chart display the 10 randomly chosen molecular function with GeneRatio and gene count. The various enzymes (ubiquitin-protein transferase, ATPase, Ras GTPase and so on), transcription coregulator, transmembrane transporter, cell cycle et,al interrelated with gene enrichment. E, Embryonic; P, Postnatal; QSC, Quiescent satellite cells; ASC, Activated satellite cells; Pro, Proliferation; Diff, Differentiation.

To investigate the interaction and the roles of lncRNAs, we conducted a GO enrichment analysis was conducted on a 5-lncRNA signature (Xist, Pvt1, Gas5, Airn, and Meg3) during skeletal muscle development (Figure 8A). This analysis revealed that these lncRNAs are significantly involved in various biological processes (BP), including muscle development, organ growth, cell proliferation, differentiation, cycle progression, migration, apoptosis, and adaptation. The top twenty BPs are depicted in Figure 8B; Supplementary Figure S2. We further calculated the GeneRatio (enrichment gene count/total gene count) and ranked these ratios to highlight the most significant associations. The bubble chart in Figure 8B; Supplementary Figure S2A shows that the five-lncRNA signature (Figure 8B; Supplementary Figure S2A; Supplementary Figure S8D-I) is strongly associated with metabolic processes, protein secretion, kinase activities, cytokine production, wound healing, DNA repair, mRNA processing, and negative regulation of the cell cycle. Additionally, the cellular component (CC) analysis indicated that these lncRNAs are enriched in several cell membranes, extracellular matrix, transport vesicles, myofibrils, actin cytoskeleton, and microtubules. The top 20 CCs are displayed in Figure 8C; Supplementary Figure S2. The bubble chart in Figure 8C; Supplementary Figure S2B illustrates notable associations with cell-cell junctions, secretory granules, transporter complexes, receptor complexes, nuclear envelopes, transcription regulator complexes, and lysosomes (Figure 8C; Supplementary Figure S2B; Supplementary Figure S2D-I). Finally, molecular function (MF) results showed enrichment in various enzyme activities, receptor ligand activities, transcription coregulator activities, ion channel activities, and transmembrane transporter activities. The top 20 MFs are shown in Figure 8D; Supplementary Figure S2. The bubble chart in Figure 8D; Supplementary Figure S2C highlights significant bindings involving actin-tubulin, Ras GTPase, phospholipids, GTP-ribonucleosides, amides, and nucleosides (Figure 8D; Supplementary Figure S2C; Supplementary Figure S2D-I). Under simulated microgravity, GO enrichment analysis showed a noticeable decrease or disappearance of BP, CC, or MF in muscle cells, as shown in Figure 8; Supplementary Figure S2. These findings indicate dynamic changes in GO functions during skeletal muscle development, underscoring the crucial role of lncRNAs in both muscle development and atrophy.

**FIGURE 9:**
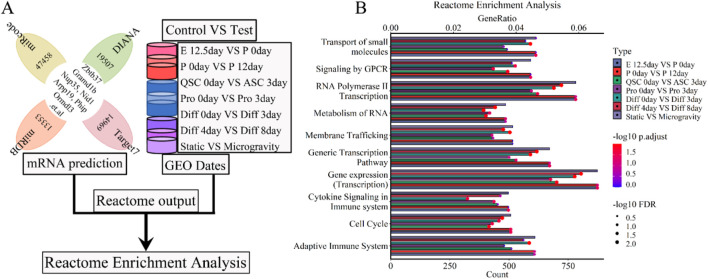
Reactome enrichment analysis for the lncRNA-miRNA-mRNA ceRNA regulatory network of various stages of skeletal muscle development. **(A)** The workflow showed that the mRNA expression of various stages of skeletal muscle development were enriched with Reactome enrichment analysis. **(B)** The bubble pattern and bar chart display the 10 randomly chosen molecular function with GeneRatio and gene count. The various enzymes (Rho GTPases, MAPK family signaling cascades, RAF/MAP kinase cascade, PIP3 activates AKT signaling, deubiquitination and so on), cell cycle, cell cycle checkpoints, metabolism, immune system et,al interrelated with gene enrichment. E, Embryonic; P, Postnatal; QSC, Quiescent satellite cells; ASC, Activated satellite cells; Pro, Proliferation; Diff, Differentiation.

We conducted a Reactome enrichment analysis to elucidate the molecular and biological functions of five lncRNAs (Xist, Pvt1, Gas5, Airn, and Meg3) skeletal muscle development. This analysis intersected predicted mRNAs with those from each phase of muscle development (Figure 9A). The results indicated that these lncRNAs are involved in muscle contraction, cell development, metabolism, and immune response. Thirty selected Reactome pathways are shown in Figures 9B; Supplementary Figure S3. We calculated and ranked the GeneRatio (enrichment gene count/total gene count) and gene count. A bubble chart illustrated the 5-lncRNA signature, showing its significant association with gene expression, small molecule transport, GPCR signaling, stress responses, Rho GTPase signaling, MAPK cascades, RAF/MAP kinase cascade, PIP3-AKT signaling, cell cycle checkpoints, and deubiquitination (Figure 9B; Supplementary Figure S3). Our used Reactome enrichment analysis revealed that cell cycle regulation, including checkpoints and mitotic phases, significantly supports muscle development (P. adjust < 0.05 and FDR < 0.1) (Figure 9B; Supplementary Figure S3). Enzymes related to RHO GTPase, RAF/MAP kinase cascade, MAPK signaling, PIP3-activated AKT signaling, and GPCR signaling also appear to be involved (P.adjust < 0.05 and FDR < 0.1) (Figure 9B; Supplementary Figure S3). Other molecular functions related to skeletal muscle development include the Adaptive Immune System, Deubiquitination, Cytokine Signaling (P.adjust < 0.05 and FDR < 0.1), and Cellular Stress Responses (P.adjust < 0.05 and FDR < 0.05). Reactome enrichment analysis (Figure 9B; Supplementary Figure S3) indicates that simulated microgravity significantly reduced or eliminated the functions and signatures of lncRNAs in muscle cells. LncRNAs Xist, Pvt1, Gas5, Airn, and Meg3 play key roles in cell cycle, metabolism, and transcription. Dysregulation in signal transduction and transcription contributes to muscle atrophy and hinders regeneration. This study highlights that these five lncRNAs significantly influence skeletal muscle development, providing valuable insights for preventing microgravity-induced+ muscle atrophy.

**FIGURE 10:**
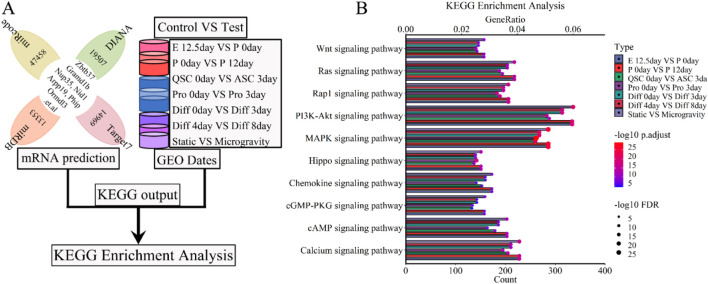
KEGG enrichment analysis for the lncRNA-miRNA-mRNA ceRNA regulatory network of various stages of skeletal muscle development. **(A)** The workflow indicated that the mRNA expression of various stages of skeletal muscle development were enriched with KEGG enrichment analysis. **(B)** The bubble pattern and bar chart show the 10 randomly chosen signaling pathway with GeneRatio and gene count. The PI3K-Akt, MAPK, Calcium, Ras, Rap signaling pathway et al. involved in regulating various stages of skeletal muscle development and muscle atrophy (waste). E, Embryonic; P, Postnatal; QSC, Quiescent satellite cells; ASC, Activated satellite cells; Pro, Proliferation; Diff, Differentiation.

Our analysis revealed the interdependence of molecular functions and signaling pathways in biological processes, where elements like lncRNA, miRNA, mRNA, proteins, and enzymes function as signaling molecules or regulators of downstream signaling pathways (Caffaratti et al., 2021). We conducted a KEGG enrichment analysis to clarify the roles of five lncRNAs (Xist, Pvt1, Gas5, Airn, Meg3) in skeletal muscle development, utilizing both predicted mRNAs and mRNAs from each developmental stage (Figure 10A). The analysis revealed enrichment in pathways including Carbon metabolism and the Cell cycle. Figure 10B; Supplementary Figure S4 depict thirty selective KEGG pathways. GeneRatio was calculated as the ratio of enriched gene count to total gene count, followed by ranking of these counts. The bubble chart (Figure 10B; Supplementary Figure S4) highlights significant associations of the 5-lncRNA signature with several signaling pathways including PI3K-Akt, MAPK, Calcium, Ras, Rap, mTOR, FoxO, TNF, TGF-beta, Hippo, and AMPK. Simulated microgravity conditions led to a notable reduction or loss of lncRNA functions and signatures in muscle cells, as evidenced by KEGG enrichment analysis (Figure 10B; Supplementary Figure S4). These results indicate that lncRNAs are instrumental in regulating skeletal muscle development and atrophy processes, including cell proliferation, differentiation, and regeneration, as differentially modulated by KEGG pathway analyses.

**FIGURE 11:**
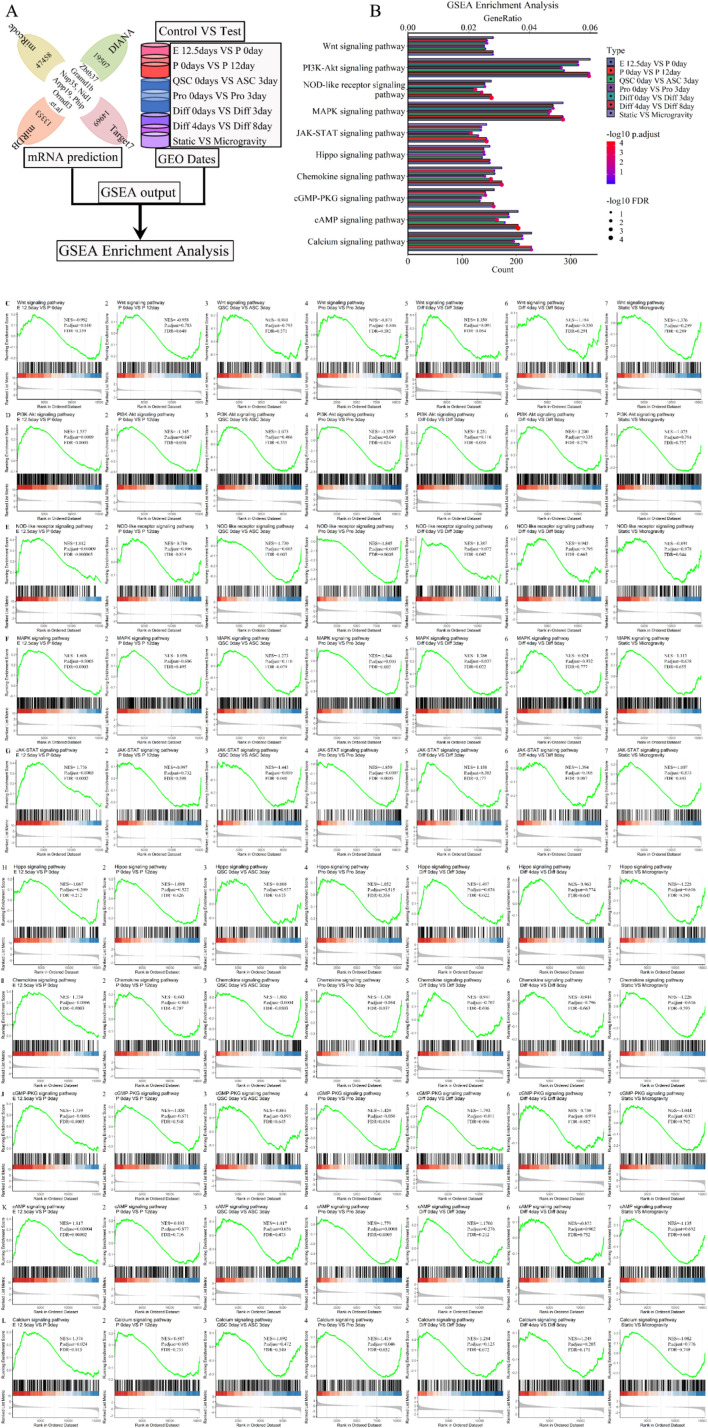
GSEA enrichment analysis for the lncRNA-miRNA-mRNA ceRNA regulatory network of various stages of skeletal muscle development. **(A)** The workflow indicated that the mRNA expression of various stages of skeletal muscle development were enriched with GSEA enrichment analysis. **(B)** The bubble pattern and bar chart show the 10 randomly chosen signaling pathway with GeneRatio and gene count. The Wnt, Hippo, Ampk, Chemokine, Apelin, PI3K-Akt, MAPK, Calcium, Ras, Rap signaling pathway et al. involved in regulating various stages of skeletal muscle development and muscle atrophy (waste). GSEA is a method that determines whether a set of genes shows differences between two biological states. The normalized enrichment score (NES) reflects the degree to which a gene set is upregulated (positive NES) or downregulated (negative NES). Corresponding p values are indicated. **(C)** GSEA plots showing enrichment of Wnt signaling pathway during skeletal muscle development and microgravity-induced muscle atrophy (waste). **(D)** GSEA plots showing enrichment of PI3K-Akt signaling pathway during skeletal muscle development and microgravity-induced muscle atrophy (waste). **(E)** GSEA plots showing enrichment of NOD-like receptor signaling pathway during skeletal muscle development and microgravity-induced muscle atrophy (waste). **(F)** GSEA plots showing enrichment of MAPK signaling pathway during skeletal muscle development and microgravity-induced muscle atrophy (waste). **(G)** GSEA plots showing enrichment of JAK-STAT signaling pathway during skeletal muscle development and microgravity-induced muscle atrophy (waste). **(H)** GSEA plots showing enrichment of Hippo signaling pathway during skeletal muscle development and microgravity-induced muscle atrophy (waste). **(I)** GSEA plots showing enrichment of Chemokine signaling pathway during skeletal muscle development and microgravity-induced muscle atrophy (waste). **(J)** GSEA plots showing enrichment of cGMP-PKG signaling pathway during skeletal muscle development and microgravity-induced muscle atrophy (waste). **(K)** GSEA plots showing enrichment of cAMP signaling pathway during skeletal muscle development and microgravity-induced muscle atrophy (waste). **(L)** GSEA plots showing enrichment of Calcium signaling pathway during skeletal muscle development and microgravity-induced muscle atrophy (waste). Normalized enrichment scores (NESs), FDR and nominal p values (Nom p value), as calculated by GSEA, are provided. skeletal muscle development stages contain E12.5 days VS P 0days, P 0days VS P 12 days, QSC 0days VS ASC 3 days, Pro 0days VS Pro 3 days, Diff 0 days VS Diff 3 days and Diff 4 days VS Diff 8 days. Microgravity-induced muscle atrophy (waste) is Static VS Microgravity. E, Embryonic; P, Postnatal; QSC, Quiescent satellite cells; ASC, Activated satellite cells; Pro, Proliferation; Diff, Differentiation.

To further validate the association of signaling pathways with the five lncRNAs (Xist, Pvt1, Gas5, Airn, and Meg3), we conducted Gene Set Enrichment Analysis (GSEA), a standard tool for assessing gene set enrichment (Wilson et al., 2010). This analysis involved the intersection of predicted mRNA from each phase of skeletal muscle development, as illustrated in the flow chart (Figure 11A). GSEA results indicated that the lncRNA signature was enriched in pathways related to muscle atrophy, focal adhesion, autophagy, actin cytoskeleton, apoptosis, ubiquitin, cell growth, carbon metabolism, and lysosome, among others. Figure 11B; Supplementary Figure S5 display 25 randomly selected pathways. We calculated and ranked the GeneRatio and gene count, then used a bubble chart to visualize the 5-lncRNA signature based on these metrics. The bubble chart shows that the 5-lncRNA signature (Figure 11B; Supplementary Figure S5) is strongly linked to various signaling pathways, including Wnt, Hippo, and others. We aimed to assess whether variations in these pathways occur at different stages of skeletal muscle development and further analyzed their association with muscle development using GSEA. We found that the Wnt, Hippo, and AMPK signaling pathways (P.adjust ≤ 0.05 and FDR ≤ 0.05) regulate differentiation from day 0 to day 3 (Figures 11B, C, H; Supplementary Figure S5A, F, W). Chemokine and Apelin pathways (P.adjust ≤ 0.005 and FDR ≤ 0.005) show regulatory functions at embryonic day 12.5 *versus* postnatal day 0, postnatal day 0 *versus* day 12, and from differentiation day 0 to day 3 (Figures 11B, I; Supplementary Figure S5A, B, D, F, V). Similar results were observed in each stage of skeletal muscle development. For instance, the cAMP (P.adjust ≤ 0.005 and FDR ≤ 0.005) and Calcium (P.adjust ≤ 0.05 and FDR ≤ 0.05) signaling pathways (Figures 10B, K, L; Supplementary Figure S5B, E) mediate cell proliferation and embryonic development. The Insulin (P.adjust ≤ 0.05 and FDR ≤ 0.05) signaling pathway was involved in cell proliferation and early differentiation (Supplementary Figure S5A, E, F, O). Additionally, the NOD-like receptor, JAK-STAT and NF-kappa B (P.adjust ≤ 0.005 and FDR ≤ 0.005) signaling pathway (Figures 11B, E, G; Supplementary Figure S5A, B, D, E, M) contributed to embryonic development, muscle regeneration and cell proliferation. Classical pathways such as PI3K-Akt and FoxO (P.adjust ≤ 0.05 and FDR ≤ 0.05) participated in postnatal growth, cell proliferation and differentiation (Figures 11B, D; Supplementary Figures S5A, B, D, E, F, P). Furthermore, the MAPK or cGMP-PKG (P.adjust ≤ 0.05 and FDR ≤ 0.05) signaling pathway was involved in embryonic development, cell proliferation and differentiation (Figures 11B, F, J; Supplementary Figure SB, E, F). Additionally, the TNF signaling pathway (P. adjust ≤ 0.005 and FDR ≤ 0.005) and Focal adhesion pathway (P. adjust ≤ 0.05 and FDR ≤ 0.05) facilitated embryonic development and postnatal growth, and were subsequently employed in cell regeneration and proliferation, differentiation respectively (Supplementary Figure S5A-C, E-G, I, Q2). The Cytokine-cytokine receptor interaction pathway (P. adjust ≤ 0.05 and FDR ≤ 0.05) influenced all processes except postnatal growth (Supplementary Figure S5A, B, D-H, R), while the Cell Cycle pathway (P. adjust ≤ 0.05 and FDR ≤ 0.05) governed the entire skeletal muscle development phase (Supplementary Figure S5A-H, T). Additional GSEA results are in Figure 11; Supplementary Figure S5. Under simulated microgravity, GSEA results showed a significant reduction or loss in the molecular functions of several signal transduction pathways involved in skeletal muscle development, including Wnt, Hippo, Chemokine, Calcium, JAK-STAT, PI3K-Akt, FoxO, MAPK, Focal adhesion, Cytokine-cytokine receptor interaction, and Cell Cycle (P. adjust ≥ 0.1 and FDR ≥ 0.1) (Figure 11B; Supplementary Figure S5). These findings demonstrate that the signaling pathway plays a role in skeletal muscle development and offers new insights and prevention strategies for microgravity-induced muscle atrophy.

## 4 Discussion

Skeletal muscle development (Salazar et al., 2016) is crucial for normal mouse growth and motor function. As the body’s largest organ, skeletal muscle regulates metabolism and energy balance under various stress conditions like microgravity and inflammation (Pant et al., 2016; Egan and Zierath, 2013). Our study integrates existing datasets to uncover novel lncRNAs related to skeletal muscle development, including Xist, Pvt1, Gas5, Airn, and Meg3, thereby fostering new hypotheses and enhancing our understanding of their molecular functions.

By analyzing 26 GEO datasets using R and Perl, we identified lncRNAs Xist, Pvt1, Gas5, Airn, and Meg3, likely due to the early publication dates (2004) and low-depth sequencing of the datasets. Notably, lncRNAs Airn, Gas5, and Xist have been implicated in skeletal muscle development and regeneration. Tiffany et al. (Dill et al., 2021) reported that lncRNA Meg3 regulates EMT to support myoblast differentiation, while Liu et al. (Liu et al., 2019b) found it promotes bovine skeletal muscle differentiation through the miR-135-MEF2C pathway. Enrico et al. (Alessio et al., 2019) suggested that lncRNA Pvt1 accelerates early muscle atrophy by affecting mitochondrial respiration, morphology, and influencing mito/autophagy, apoptosis, and myofiber size *in vivo*. Our findings, derived from analyzing 26 datasets, align with previous studies and confirm the reliability of our results. Despite the risk of filtering out many lncRNAs and potential artefacts, our integrated analysis overcomes these limitations by pooling samples, re-annotating, analyzing all datasets, and removing batch effects. This approach minimizes single-analysis risk and human interference, allowing us to map the global response of lncRNAs Xist, Pvt1, Gas5, Airn, and Meg3 to various skeletal muscle development phases.

Studies on lncRNAs primarily focus on their ceRNA networks with miRNAs and mRNAs, influencing cell growth, development, and diseases such as muscle atrophy and cancer (Winkle et al., 2021; Thomson and Dinger, 2016; Qi et al., 2020; Matsui and Corey, 2017). Under microgravity, significant changes in lncRNAs provide new insights into skeletal muscle development. We identified relevant lncRNAs and constructed a ceRNA network by predicting their interactions with miRNAs and mRNAs. The predicted miRNAs, known to regulate muscle function (Horak et al., 2016), were also observed in a microgravity-induced muscle regeneration model, including miR-206, miR-133, miR-499, and miR-23-5p (Supplementary Figure S1). We examined several ceRNA regulatory networks in muscle tissue or cells, including lncRNA Xist/miR-499-5p/znrf2, lncRNA Pvt1/miR-140-5p/Co14a2, lncRNA Meg3/miR-330-5p/Fads1, lncRNA Gas5/miR-362-3p/Mcf21, and lncRNA Airn/miR-342-3p/Hoxb8 (Figure 7C; Supplementary Table S1). Cheng et al. (Cheng et al., 2020) and Wang et al. (Wang et al., 2021). showed that lncRNA Xist/miR-126 enhances cell proliferation and glucose metabolism via the IRS1/PI3K/Akt pathway in glioma. Liao et al. (Liao et al., 2019) and Wang et al. (Wang et al., 2021) found that lncRNA Xist/miR-17-5P/AHNAK inhibits OPLL by activating the BMP2 signaling pathway in primary human ligament fibroblast cells. Xiong et al. (Xiong et al., 2021) and Wang et al. (Wang et al., 2021) indicated that lncRNA Xist/miR-486-5p/GAB2 promotes cerebral ischemia-reperfusion injury in SH-SY5Y cells. Similar studies have been conducted on other lncRNAs. Wu et al. (Wu et al., 2019) found that lncRNA Gas5/miR-335/ROCK1 alleviates myocardial ischemia/reperfusion injury via the AKT/GSK-3β axis. Chi et al. (Chi et al., 2019) demonstrated that lncRNA Gas5/miR-455-5p/SOCS3 promotes M1 macrophage polarization by activating the JAK2/STAT3 pathway in childhood pneumonia. Xi et al. (Xi et al., 2020) showed that lncRNA Pvt1/miR-148/RAB34 enhances cell proliferation and migration in NSCLC progression. Our findings align with previous research (Figure 7C; Supplementary Table S1) on constructing a ceRNA regulatory network using R and Perl, validating our analysis. Our results suggest that lncRNAs likely collaborate with miRNAs, mRNAs, or other cytokines, rather than acting independently, in regulating physiological and biochemical functions.

The ceRNA regulatory network of lncRNA-miRNA-mRNA underpins its cellular regulatory role. Our analysis shows it primarily engages in biological processes like muscle and organ development, cell proliferation and differentiation, wound response, cell cycle, and protein secretion regulation (see Figures 8, 9; Supplementary Figure S2, 3). This activated various enzymes, proteins, transcription pathways, and cell cytokines, including ATPase, GTPase, PIP3 kinase, MAPK kinase, GPCR kinase, ion channel proteins, and cell cycle proteins, across different organelle membranes, endoplasmic reticulum, cytoskeleton, cell adhesion sites, and both inside and outside the nucleus (Figure 8; Figure 9; Supplementary Figure S2; Supplementary Figure S3). Additionally, studies (Pedersen and Febbraio, 2012; Korotkova and Lundberg, 2014; von Haehling et al., 2017; Cohen et al., 2015) indicate that muscle cells secrete cytokines and proteases that influence skeletal muscle development by activating downstream signaling molecules. Research by Chen et al. (Chen et al., 2020a; Chen et al., 2020b), Wang et al. (Wang et al., 2019b), and Luo et al. (Luo et al., 2021) further shows that the ceRNA regulatory network of lncRNA-miRNA-mRNA supports muscle development processes such as cell proliferation, differentiation, regeneration, and atrophy by regulating downstream signaling pathways. Our findings closely matched current data, validating our GO and reactome analysis. Ultimately, we identified the ceRNA regulatory network of lncRNA-miRNA-mRNA as a key facilitator of skeletal muscle development in terms of biological processes, cellular components, and molecular functions.

Signaling pathways within the ceRNA regulatory network of lncRNA-miRNA-mRNA are vital for controlling cell growth, proliferation, differentiation, and energy metabolism. Our analysis identified several key pathways—cell cycle, PI3K/Akt, TNF, Wnt, Insulin, IGF1, MAPK, NF-κB, Apoptosis, Focal adhesion, Calcium, TGF-beta, cytokine-cytokine receptor interaction, neuroactive ligand-receptor interaction, chemokine, actin cytoskeleton regulation, and protein processing in the endoplasmic reticulum—that may impact muscle diseases and skeletal muscle development (Figures 10, 11; Supplementary Figure S4, 5). Previous research has shown that pathways such as PI3K/Akt, TNF, Wnt, Insulin, IGF1, MAPK, and NF-κB regulate skeletal muscle development (Egan and Zierath, 2013; Braun and Gautel, 2011; Egan et al., 2016). Misregulation of these pathways can lead to muscle-related conditions like sarcopenia and atrophy, often caused by cancer, diabetes, chronic nephritis, and microgravity (von Haehling et al., 2017; Cohen et al., 2015; Wang and Mitch, 2014; Sharma et al., 2015; Han and Mitch, 2011). Our study supports these findings, revealing that these pathways exhibit contrasting roles at different developmental stages (Figures 10, 11; Supplementary Figure S4, 5). For example, the PI3K/Akt pathway is active during embryonic development, postnatal growth, and muscle cell proliferation but inactive at other phases. Conversely, the MAPK pathway is upregulated during embryonic development, cell proliferation, and differentiation but downregulated at other times.

The Hippo, AMPK, NF-κB, and cAMP signaling pathways show varied activation during different stages of muscle development, while the cell cycle pathway consistently supports the entire process (Figures 10, 11; Supplementary Figure S4, 5). The AMPK pathway’s (Thomson, 2018) role in muscle development is hindered under microgravity, potentially causing muscle atrophy (Figures 10, 11; Supplementary Figure S4, 5). Studies have shown that this pathway negatively affects muscle size, hypertrophy, and regeneration (Thomson, 2018). The Hippo signaling network is crucial for myogenesis, regeneration, muscular dystrophy, and rhabdomyosarcoma (Wackerhage et al., 2014; Watt et al., 2018), (Wackerhage et al., 2014; Watt et al., 2018). Additionally, NF-κB and cAMP signaling pathways regulate muscle development through cell proliferation and differentiation (Peterson et al., 2011; Ravnskjaer et al., 2016) (Peterson et al., 2011; Ravnskjaer et al., 2016), while PI3K/Akt and MAPK also play significant regulatory roles (Cohen et al., 2015; Segales et al., 2016), (Cohen et al., 2015; Segales et al., 2016). The cell cycle pathway regulates cell growth, muscle development, proliferation, differentiation, and pathology (Adhikari et al., 2019; Bischoff, 1990; Ciemerych et al., 2011; Halevy et al., 1995). Our KEGG and GSEA enrichment analysis confirmed these findings, enhancing the reliability of our results.

We identified distinct roles for lncRNAs Xist, Pvt1, Gas5, Airn, and Meg3 in muscle development and related pathologies. Demonstrating that lncRNAs can protect against muscle atrophy caused by simulated weightlessness opens new research possibilities. More data and clinical experiments are needed to test our hypothesis, along with comprehensive technical analyses using multi-omics technologies and single-cell RNA sequencing to uncover functional changes in noncoding RNA, proteins, proteases, cytokinesis, and kinase in muscle development. Our multilevel data-integration approach, using extensive mouse studies and Affymetrix chip datasets analyzed with R or Perl, will advance noncoding RNA research and develop new lncRNA-based therapies.

## 5 Conclusion

We demonstrated that skeletal muscle development and atrophy can be precisely controlled using a 5-lncRNA signature (Xist, Pvt1, Gas5, Airn, Meg3). Our findings suggest that well-regulated signaling pathways are essential for stable muscle development. However, further validation in larger independent studies is necessary to confirm our results.

## Data Availability

The datasets presented in this study can be found in online repositories. The names of the repository/repositories and accession number(s) can be found in the article/Supplementary Material.

## Abbreviations

LncRNAs, long noncoding RNAs; GEO, Gene Expression Omnibus; DEG, differentially expressed gene; DMD, Duchenne Muscular Dystrophy; GO, Gene Ontology; BP, biological processes; CC, cellular component; MF, molecular function; KEGG, Kyoto Encyclopedia of Genes and Genomes; GeneRatio, enrichment gene count/total gene count; ceRNA, Competing Endogenous RNA; Xist, X-inactive specific transcript.

## References

[B1] Adhikari A. Mainali P. Davie J. K. (2019). JARID2 and the PRC2 complex regulate the cell cycle in skeletal muscle. J. Biol. Chem. 294 (51), 19451–19464. 10.1074/jbc.ra119.010060 31578284 PMC6926451

[B2] Agarwal V. Bell G. W. Nam J. W. Bartel D. P. (2015). Predicting effective microRNA target sites in mammalian mRNAs. Elife 4, e05005. 10.7554/elife.05005 26267216 PMC4532895

[B3] Aguilar C. A. Pop R. Shcherbina A. Watts A. Matheny R. Jr. Cacchiarelli D. (2016). Transcriptional and chromatin dynamics of muscle regeneration after severe trauma. Stem Cell Rep. 7 (5), 983–997. 10.1016/j.stemcr.2016.09.009 PMC5106515 27773702

[B4] Alessio E. Buson L. Chemello F. Peggion C. Grespi F. Martini P. (2019). Single cell analysis reveals the involvement of the long non-coding RNA Pvt1 in the modulation of muscle atrophy and mitochondrial network. Nucleic Acids Res. 47 (4), 1653–1670. 10.1093/nar/gkz007 30649422 PMC6393313

[B5] Ballarino M. Morlando M. Fatica A. Bozzoni I. (2016). Non-coding RNAs in muscle differentiation and musculoskeletal disease. J. Clin. Invest 126 (6), 2021–2030. 10.1172/jci84419 27249675 PMC4887180

[B6] Bentzinger C. F. Wang Y. X. Rudnicki M. A. (2012). Building muscle: molecular regulation of myogenesis. Cold Spring Harb. Perspect. Biol. 4 (2), a008342. 10.1101/cshperspect.a008342 22300977 PMC3281568

[B7] Bischoff R. (1990). Cell cycle commitment of rat muscle satellite cells. J. Cell Biol. 111 (1), 201–207. 10.1083/jcb.111.1.201 2365732 PMC2116175

[B8] Braun T. Gautel M. (2011). Transcriptional mechanisms regulating skeletal muscle differentiation, growth and homeostasis. Nat. Rev. Mol. Cell Biol. 12 (6), 349–361. 10.1038/nrm3118 21602905

[B9] Bricchi I. Bertea C. M. Occhipinti A. Paponov I. A. Maffei M. E. (2012). Dynamics of membrane potential variation and gene expression induced by Spodoptera littoralis, *Myzus persicae*, and *Pseudomonas syringae* in Arabidopsis. PLoS One 7 (10), e46673. 10.1371/journal.pone.0046673 23118859 PMC3484130

[B10] Burzyn D. Kuswanto W. Kolodin D. Shadrach J. Cerletti M. Jang Y. (2013). A special population of regulatory T cells potentiates muscle repair. Cell 155 (6), 1282–1295. 10.1016/j.cell.2013.10.054 24315098 PMC3894749

[B11] Butchart L. C. Fox A. Shavlakadze T. Grounds M. D. (2016). The long and short of non-coding RNAs during post-natal growth and differentiation of skeletal muscles: focus on lncRNA and miRNAs. Differentiation 92 (5), 237–248. 10.1016/j.diff.2016.05.003 27292314

[B12] Caffaratti C. Plazy C. Mery G. Tidjani A. R. Fiorini F. Thiroux S. (2021). What we know so far about the metabolite-mediated microbiota-intestinal immunity dialogue and how to hear the sound of this crosstalk. Metabolites 11 (6), 406. 10.3390/metabo11060406 34205653 PMC8234899

[B13] Chal J. Pourquie O. (2017). Making muscle: skeletal myogenesis *in vivo* and *in vitro* . Development 144 (12), 2104–2122. 10.1242/dev.151035 28634270

[B14] Chen I. H. Huber M. Guan T. Bubeck A. Gerace L. (2006). Nuclear envelope transmembrane proteins (NETs) that are up-regulated during myogenesis. BMC Cell Biol. 7 (38), 38–16. 10.1186/1471-2121-7-38 17062158 PMC1635557

[B15] Chen L. L. (2016). Linking long noncoding RNA localization and function. Trends Biochem. Sci. 41 (9), 761–772. 10.1016/j.tibs.2016.07.003 27499234

[B16] Chen R. Jiang T. She Y. Xie S. Zhou S. Li C. (2018a). Comprehensive analysis of lncRNAs and mRNAs with associated co-expression and ceRNA networks in C2C12 myoblasts and myotubes. Gene 647, 164–173. 10.1016/j.gene.2018.01.039 29331478

[B17] Chen R. Jiang T. She Y. Xie S. Zhou S. Li C. (2018b). Comprehensive analysis of lncRNAs and mRNAs with associated co-expression and ceRNA networks in C2C12 myoblasts and myotubes. Gene 647 (39), 164–173. 10.1016/j.gene.2018.01.039 29331478

[B18] Chen R. Lei S. Jiang T. She Y. Shi H. (2020a). Regulation of skeletal muscle atrophy in cachexia by MicroRNAs and long non-coding RNAs. Front. Cell Dev. Biol. 8 (2020), 577010–577022. 10.3389/fcell.2020.577010 33043011 PMC7523183

[B19] Chen R. Lei S. Jiang T. Zeng J. Zhou S. She Y. (2020b). Roles of lncRNAs and circRNAs in regulating skeletal muscle development. Acta Physiol. (Oxf) 228 (2), e13356. 10.1111/apha.13356 31365949

[B20] Chen X. He L. Zhao Y. Li Y. Zhang S. Sun K. (2017). Malat1 regulates myogenic differentiation and muscle regeneration through modulating MyoD transcriptional activity. Cell Discov. 3 (2017), 17002–17024. 10.1038/celldisc.2017.2 28326190 PMC5348715

[B21] Chen Y. Wang X. (2020). miRDB: an online database for prediction of functional microRNA targets. Nucleic Acids Res. 48 (D1), D127–D131. 10.1093/nar/gkz757 31504780 PMC6943051

[B22] Cheng Q. Chen X. Wu H. Du Y. (2021). Three hematologic/immune system-specific expressed genes are considered as the potential biomarkers for the diagnosis of early rheumatoid arthritis through bioinformatics analysis. J. Transl. Med. 19 (1), 18–32. 10.1186/s12967-020-02689-y 33407587 PMC7789535

[B23] Cheng Z. Luo C. Guo Z. (2020). LncRNA-XIST/microRNA-126 sponge mediates cell proliferation and glucose metabolism through the IRS1/PI3K/Akt pathway in glioma. J. Cell Biochem. 121 (3), 2170–2183. 10.1002/jcb.29440 31680298

[B24] Chi X. Ding B. Zhang L. Zhang J. Wang J. Zhang W. (2019). lncRNA GAS5 promotes M1 macrophage polarization via miR-455-5p/SOCS3 pathway in childhood pneumonia. J. Cell Physiol. 234 (8), 13242–13251. 10.1002/jcp.27996 30584669 PMC13483271

[B25] Chini M. Hanganu-Opatz I. L. (2021). Prefrontal cortex development in health and disease: lessons from rodents and humans. Trends Neurosci. 44 (3), 227–240. 10.1016/j.tins.2020.10.017 33246578

[B26] Cho I. J. Lui P. P. Obajdin J. Riccio F. Stroukov W. Willis T. L. (2019). Mechanisms, hallmarks, and implications of stem cell quiescence. Stem Cell Rep. 12 (6), 1190–1200. 10.1016/j.stemcr.2019.05.012 PMC6565921 31189093

[B27] Ciemerych M. A. Archacka K. Grabowska I. Przewoźniak M. (2011). Cell cycle regulation during proliferation and differentiation of mammalian muscle precursor cells. Results Probl. Cell Differ. 53, 473–527. 10.1007/978-3-642-19065-0_20 21630157

[B28] Ciemerych M. A. Sicinski P. (2005). Cell cycle in mouse development. Oncogene 24 (17), 2877–2898. 10.1038/sj.onc.1208608 15838522

[B29] Cohen S. Nathan J. A. Goldberg A. L. (2015). Muscle wasting in disease: molecular mechanisms and promising therapies. Nat. Rev. Drug Discov. 14 (1), 58–74. 10.1038/nrd4467 25549588

[B30] Dill T. L. Carroll A. Pinheiro A. Gao J. Naya F. J. (2021). The long noncoding RNA Meg3 regulates myoblast plasticity and muscle regeneration through epithelial-mesenchymal transition. Development 148 (2), dev194027. 10.1242/dev.194027 33298462

[B31] Dumont N. A. Bentzinger C. F. Sincennes M. C. Rudnicki M. A. (2015). Satellite cells and skeletal muscle regeneration. Compr. Physiol. 5 (3), 1027–1059. 10.1002/cphy.c140068 26140708

[B32] d'Ydewalle C. Ramos D. M. Pyles N. J. Ng S. Y. Gorz M. Pilato C. M. (2017). The antisense transcript SMN-AS1 regulates SMN expression and is a novel therapeutic target for spinal muscular atrophy. Neuron 93 (1), 66–79. 10.1016/j.neuron.2016.11.033 28017471 PMC5223741

[B33] Egan B. Hawley J. A. Zierath J. R. (2016). SnapShot: exercise metabolism. Cell Metab. 24 (2), 342–342.e1. 10.1016/j.cmet.2016.07.013 27508878

[B34] Egan B. Zierath J. R. (2013). Exercise metabolism and the molecular regulation of skeletal muscle adaptation. Cell Metab. 17 (2), 162–184. 10.1016/j.cmet.2012.12.012 23395166

[B35] Farina N. H. Hausburg M. Betta N. D. Pulliam C. Srivastava D. Cornelison D. (2012). A role for RNA post-transcriptional regulation in satellite cell activation. Skelet. Muscle 2 (1), 21. 10.1186/2044-5040-2-21 23046558 PMC3563611

[B36] Frangini M. Franzolin E. Chemello F. Laveder P. Romualdi C. Bianchi V. (2013). Synthesis of mitochondrial DNA precursors during myogenesis, an analysis in purified C2C12 myotubes. J. Biol. Chem. 288 (8), 5624–5635. 10.1074/jbc.m112.441147 23297407 PMC3581417

[B37] Fukada S. Uezumi A. Ikemoto M. Masuda S. Segawa M. Tanimura N. (2007). Molecular signature of quiescent satellite cells in adult skeletal muscle. Stem Cells 25 (10), 2448–2459. 10.1634/stemcells.2007-0019 17600112

[B38] Garcia-Prat L. Martinez-Vicente M. Perdiguero E. Ortet L. Rodríguez-Ubreva J. Rebollo E. (2016). Autophagy maintains stemness by preventing senescence. Nature 529 (7584), 37–42. 10.1038/nature16187 26738589

[B39] Garrett-Bakelman F. E. Darshi M. Green S. J. Gur R. C. Lin L. Macias B. R. (2019). The NASA Twins Study: a multidimensional analysis of a year-long human spaceflight. Science 364 (6436), eaau8650. 10.1126/science.aau8650 30975860 PMC7580864

[B40] Genchi G. G. Degl'Innocenti A. Martinelli C. Battaglini M. De Pasquale D. Prato M. (2021). Cerium oxide nanoparticle administration to skeletal muscle cells under different gravity and radiation conditions. ACS Appl. Mater Interfaces 13 (34), 40200–40213. 10.1021/acsami.1c14176 34410709 PMC8414486

[B41] Goel A. J. Rieder M. K. Arnold H. H. Radice G. L. Krauss R. S. (2017). Niche cadherins control the quiescence-to-activation transition in muscle stem cells. Cell Rep. 21 (8), 2236–2250. 10.1016/j.celrep.2017.10.102 29166613 PMC5702939

[B42] Goncalves T. J. M. Armand A. S. (2017). Non-coding RNAs in skeletal muscle regeneration. Noncoding RNA Res. 2 (1), 56–67. 10.1016/j.ncrna.2017.03.003 30159421 PMC6096429

[B43] Gong C. Li Z. Ramanujan K. Clay I. Zhang Y. Lemire-Brachat S. (2015). A long non-coding RNA, LncMyoD, regulates skeletal muscle differentiation by blocking IMP2-mediated mRNA translation. Dev. Cell 34 (2), 181–191. 10.1016/j.devcel.2015.05.009 26143994

[B44] Gupta A. Lutolf M. P. Hughes A. J. Sonnen K. F. (2021). Bioengineering *in vitro* models of embryonic development. Stem Cell Rep. 16 (5), 1104–1116. 10.1016/j.stemcr.2021.04.005 PMC8185467 33979597

[B45] Halevy O. Novitch B. G. Spicer D. B. Skapek S. X. Rhee J. Hannon G. J. (1995). Correlation of terminal cell cycle arrest of skeletal muscle with induction of p21 by MyoD. Science 267 (5200), 1018–1021. 10.1126/science.7863327 7863327

[B46] Han H. Q. Mitch W. E. (2011). Targeting the myostatin signaling pathway to treat muscle wasting diseases. Curr. Opin. Support Palliat. Care 5 (4), 334–341. 10.1097/spc.0b013e32834bddf9 22025090 PMC3273421

[B47] Han X. Yang F. Cao H. Liang Z. (2015). Malat1 regulates serum response factor through miR-133 as a competing endogenous RNA in myogenesis. FASEB J. 29 (7), 3054–3064. 10.1096/fj.14-259952 25868726

[B48] Hemberger M. Hanna C. W. Dean W. (2020). Mechanisms of early placental development in mouse and humans. Nat. Rev. Genet. 21 (1), 27–43. 10.1038/s41576-019-0169-4 31534202

[B49] Hnia K. Clausen T. Moog-Lutz C. (2019). Shaping striated muscles with ubiquitin proteasome system in health and disease. Trends Mol. Med. 25 (9), 760–774. 10.1016/j.molmed.2019.05.008 31235369

[B50] Horak M. Novak J. Bienertova-Vasku J. (2016). Muscle-specific microRNAs in skeletal muscle development. Dev. Biol. 410 (1), 1–13. 10.1016/j.ydbio.2015.12.013 26708096

[B51] Huang C. Wu S. Ji H. Yan X. Xie Y. Murai S. (2017). Identification of XBP1-u as a novel regulator of the MDM2/p53 axis using an shRNA library. Sci. Adv. 3 (10), e1701383. 10.1126/sciadv.1701383 29057323 PMC5647124

[B52] Hupkes M. Sotoca A. M. Hendriks J. M. van Zoelen E. J. Dechering K. J. (2014). MicroRNA miR-378 promotes BMP2-induced osteogenic differentiation of mesenchymal progenitor cells. BMC Mol. Biol. 15 (1), 1–15. 10.1186/1471-2199-15-1 24467925 PMC3905160

[B53] Ito K. Murphy D. (2013). Application of ggplot2 to pharmacometric graphics. CPT Pharmacometrics Syst. Pharmacol. 2 (2013), 1–16. 10.1038/psp.2013.56 PMC3817376 24132163

[B54] Jassal B. Matthews L. Viteri G. Gong C. Lorente P. Fabregat A. (2020). The reactome pathway knowledgebase. Nucleic Acids Res. 48 (D1), D498-D503–D503. 10.1093/nar/gkz1031 31691815 PMC7145712

[B55] Jeggari A. Marks D. S. Larsson E. (2012). miRcode: a map of putative microRNA target sites in the long non-coding transcriptome. Bioinformatics 28 (15), 2062–2063. 10.1093/bioinformatics/bts344 22718787 PMC3400968

[B56] Jin J. J. Lv W. Xia P. Xu Z. Y. Zheng A. D. Wang X. J. (2018). Long noncoding RNA SYISL regulates myogenesis by interacting with polycomb repressive complex 2. Proc. Natl. Acad. Sci. U. S. A. 115 (42), E9802-E9811–E9811. 10.1073/pnas.1801471115 30279181 PMC6196504

[B57] Kallen A. N. Zhou X. B. Xu J. Qiao C. Ma J. Yan L. (2013). The imprinted H19 lncRNA antagonizes let-7 microRNAs. Mol. Cell 52 (1), 101–112. 10.1016/j.molcel.2013.08.027 24055342 PMC3843377

[B58] Karagkouni D. Paraskevopoulou M. D. Chatzopoulos S. Vlachos I. S. Tastsoglou S. Kanellos I. (2018). DIANA-TarBase v8: a decade-long collection of experimentally supported miRNA-gene interactions. Nucleic Acids Res. 46 (D1), D239–D245. 10.1093/nar/gkx1141 29156006 PMC5753203

[B59] Karagkouni D. Paraskevopoulou M. D. Tastsoglou S. Skoufos G. Karavangeli A. Pierros V. (2020). DIANA-LncBase v3: indexing experimentally supported miRNA targets on non-coding transcripts. Nucleic Acids Res. 48 (D1), D101-D110–D110. 10.1093/nar/gkz1036 31732741 PMC7145509

[B60] Korotkova M. Lundberg I. E. (2014). The skeletal muscle arachidonic acid cascade in health and inflammatory disease. Nat. Rev. Rheumatol. 10 (5), 295–303. 10.1038/nrrheum.2014.2 24468934

[B61] Kostallari E. Baba-Amer Y. Alonso-Martin S. Ngoh P. Relaix F. Lafuste P. (2015). Pericytes in the myovascular niche promote post-natal myofiber growth and satellite cell quiescence. Development 142 (7), 1242–1253. 10.1242/dev.115386 25742797

[B62] Lander A. D. (2011). Pattern, growth, and control. Cell 144 (6), 955–969. 10.1016/j.cell.2011.03.009 21414486 PMC3128888

[B63] Latroche C. Weiss-Gayet M. Muller L. Gitiaux C. Leblanc P. Liot S. (2017). Coupling between myogenesis and angiogenesis during skeletal muscle regeneration is stimulated by restorative macrophages. Stem Cell Rep. 9 (6), 2018–2033. 10.1016/j.stemcr.2017.10.027 PMC5785732 29198825

[B64] Lattin J. E. Schroder K. Su A. I. Walker J. R. Zhang J. Wiltshire T. (2008). Expression analysis of G Protein-Coupled Receptors in mouse macrophages. Immunome Res. 4 (5), 5–13. 10.1186/1745-7580-4-5 18442421 PMC2394514

[B65] Lee Y. S. Huynh T. V. Lee S. J. (1985)2016). Paracrine and endocrine modes of myostatin action. J. Appl. Physiol. 120 (6), 592–598. 10.1152/japplphysiol.00874.2015 PMC4796182 26769954

[B66] Legnini I. Morlando M. Mangiavacchi A. Fatica A. Bozzoni I. (2014). A feedforward regulatory loop between HuR and the long noncoding RNA linc-MD1 controls early phases of myogenesis. Mol. Cell 53 (3), 506–514. 10.1016/j.molcel.2013.12.012 24440503 PMC3919156

[B67] Lei F. Zhang H. Xie X. (2019). Comprehensive analysis of an lncRNA-miRNA-mRNA competing endogenous RNA network in pulpitis. PeerJ 7 (2019), e7135. 10.7717/peerj.7135 31304055 PMC6609876

[B68] Li J. H. Liu S. Zhou H. Qu L. H. Yang J. H. (2014). starBase v2.0: decoding miRNA-ceRNA, miRNA-ncRNA and protein-RNA interaction networks from large-scale CLIP-Seq data. Nucleic Acids Res. 42 (Database issue), D92–D97. 10.1093/nar/gkt1248 24297251 PMC3964941

[B69] Li Y. Chen X. Sun H. Wang H. (2018). Long non-coding RNAs in the regulation of skeletal myogenesis and muscle diseases. Cancer Lett. 417 (2018), 58–64. 10.1016/j.canlet.2017.12.015 29253523

[B70] Li Z. Cai B. Abdalla B. A. Zhu X. Zheng M. Han P. (2019). LncIRS1 controls muscle atrophy via sponging miR-15 family to activate IGF1-PI3K/AKT pathway. J. Cachexia Sarcopenia Muscle 10 (2), 391–410. 10.1002/jcsm.12374 30701698 PMC6463472

[B71] Liao X. Tang D. Yang H. Chen Y. Chen D. Jia L. (2019). Long non-coding RNA XIST may influence cervical ossification of the posterior longitudinal ligament through regulation of miR-17-5P/AHNAK/BMP2 signaling pathway. Calcif. Tissue Int. 105 (6), 670–680. 10.1007/s00223-019-00608-y 31511959

[B72] Liu J. Yan Y. Xi B. Huang G. Mi J. (2019a). Skeletal muscle reference for Chinese children and adolescents. J. Cachexia Sarcopenia Muscle 10 (1), 155–164. 10.1002/jcsm.12361 30499245 PMC6438334

[B73] Liu M. Li B. Peng W. Ma Y. Huang Y. Lan X. (2019b). LncRNA-MEG3 promotes bovine myoblast differentiation by sponging miR-135. J. Cell Physiol. 234 (10), 18361–18370. 10.1002/jcp.28469 30887511

[B74] Liu N. Garry G. A. Li S. Bezprozvannaya S. Sanchez-Ortiz E. Chen B. (2017a). A Twist2-dependent progenitor cell contributes to adult skeletal muscle. Nat. Cell Biol. 19 (3), 202–213. 10.1038/ncb3477 28218909 PMC5332283

[B75] Liu W. Nanetti A. Cheong S. A. (2017b). Knowledge evolution in physics research: an analysis of bibliographic coupling networks. PLoS One 12 (9), e0184821. 10.1371/journal.pone.0184821 28922427 PMC5602641

[B76] Liu Y. Chu A. Chakroun I. Islam U. Blais A. (2010). Cooperation between myogenic regulatory factors and SIX family transcription factors is important for myoblast differentiation. Nucleic Acids Res. 38 (20), 6857–6871. 10.1093/nar/gkq585 20601407 PMC2978361

[B77] Lu L. Sun K. Chen X. Zhao Y. Wang L. Zhou L. (2013). Genome-wide survey by ChIP-seq reveals YY1 regulation of lincRNAs in skeletal myogenesis. EMBO J. 32 (19), 2575–2588. 10.1038/emboj.2013.182 23942234 PMC3791367

[B78] Lukjanenko L. Brachat S. Pierrel E. Lach-Trifilieff E. Feige J. N. (2013). Genomic profiling reveals that transient adipogenic activation is a hallmark of mouse models of skeletal muscle regeneration. PLoS One 8 (8), e71084. 10.1371/journal.pone.0071084 23976982 PMC3744575

[B79] Luo H. Lv W. Tong Q. Jin J. Xu Z. Zuo B. (2021). Functional non-coding RNA during embryonic myogenesis and postnatal muscle development and disease. Front. Cell Dev. Biol. 9 (2021), 628339–628343. 10.3389/fcell.2021.628339 33585483 PMC7876409

[B80] Ma Q. Chirn G. W. Szustakowski J. D. Bakhtiarova A. Kosinski P. A. Kemp D. (2008). Uncovering mechanisms of transcriptional regulations by systematic mining of cis regulatory elements with gene expression profiles. BioData Min. 1 (1), 4–10. 10.1186/1756-0381-1-4 18822150 PMC2553773

[B81] Mahdessian D. Cesnik A. J. Gnann C. Danielsson F. Stenström L. Arif M. (2021). Spatiotemporal dissection of the cell cycle with single-cell proteogenomics. Nature 590 (7847), 649–654. 10.1038/s41586-021-03232-9 33627808

[B82] Matsui M. Corey D. R. (2017). Non-coding RNAs as drug targets. Nat. Rev. Drug Discov. 16 (3), 167–179. 10.1038/nrd.2016.117 27444227 PMC5831170

[B83] Matsumoto A. Pasut A. Matsumoto M. Yamashita R. Fung J. Monteleone E. (2017). mTORC1 and muscle regeneration are regulated by the LINC00961-encoded SPAR polypeptide. Nature 541 (7636), 228–232. 10.1038/nature21034 28024296

[B84] Metsalu T. Vilo J. (2015). ClustVis: a web tool for visualizing clustering of multivariate data using Principal Component Analysis and heatmap. Nucleic Acids Res. 43 (W1), W566–W570. 10.1093/nar/gkv468 25969447 PMC4489295

[B85] Militello G. Hosen M. R. Ponomareva Y. Gellert P. Weirick T. John D. (2018). A novel long non-coding RNA Myolinc regulates myogenesis through TDP-43 and Filip1. J. Mol. Cell Biol. 10 (2), 102–117. 10.1093/jmcb/mjy025 29618024 PMC7191624

[B86] Mourikis P. Gopalakrishnan S. Sambasivan R. Tajbakhsh S. (2012). Cell-autonomous Notch activity maintains the temporal specification potential of skeletal muscle stem cells. Development 139 (24), 4536–4548. 10.1242/dev.084756 23136394

[B87] Ogawa R. Ma Y. Yamaguchi M. Ito T. Watanabe Y. Ohtani T. (2015). Doublecortin marks a new population of transiently amplifying muscle progenitor cells and is required for myofiber maturation during skeletal muscle regeneration. Development 142 (1), 810–861. 10.1242/dev.122317 25480916

[B88] Pant M. Bal N. C. Periasamy M. (2016). Sarcolipin: a key thermogenic and metabolic regulator in skeletal muscle. Trends Endocrinol. Metab. 27 (12), 881–892. 10.1016/j.tem.2016.08.006 27637585 PMC5424604

[B89] Peault B. Rudnicki M. Torrente Y. Cossu G. Tremblay J. P. Partridge T. (2007). Stem and progenitor cells in skeletal muscle development, maintenance, and therapy. Mol. Ther. 15 (5), 867–877. 10.1038/mt.sj.6300145 17387336

[B90] Pedersen B. K. Febbraio M. A. (2012). Muscles, exercise and obesity: skeletal muscle as a secretory organ. Nat. Rev. Endocrinol. 8 (8), 457–465. 10.1038/nrendo.2012.49 22473333

[B91] Peterson J. M. Bakkar N. Guttridge D. C. (2011). NF-κB signaling in skeletal muscle health and disease. Curr. Top. Dev. Biol. 96 (2011), 85–119. 10.1016/B978-0-12-385940-2.00004-8 21621068

[B92] Pillon N. J. Gabriel B. M. Dollet L. Smith J. A. B. Sardón Puig L. Botella J. (2020). Transcriptomic profiling of skeletal muscle adaptations to exercise and inactivity. Nat. Commun. 11 (1), 470–484. 10.1038/s41467-019-13869-w 31980607 PMC6981202

[B93] Plikus M. V. Wang X. Sinha S. Forte E. Thompson S. M. Herzog E. L. (2021). Fibroblasts: origins, definitions, and functions in health and disease. Cell 184 (15), 3852–3872. 10.1016/j.cell.2021.06.024 34297930 PMC8566693

[B94] Ponnusamy M. Li P. F. Wang K. (2017). Understanding cardiomyocyte proliferation: an insight into cell cycle activity. Cell Mol. Life Sci. 74 (6), 1019–1034. 10.1007/s00018-016-2375-y 27695872 PMC11107761

[B95] Ponting C. P. Oliver P. L. Reik W. (2009). Evolution and functions of long noncoding RNAs. Cell 136 (4), 629–641. 10.1016/j.cell.2009.02.006 19239885

[B96] Qi X. Lin Y. Chen J. Shen B. (2020). Decoding competing endogenous RNA networks for cancer biomarker discovery. Brief. Bioinform 21 (2), 441–457. 10.1093/bib/bbz006 30715152

[B97] Rajan S. Chu Pham Dang H. Djambazian H. Zuzan H. Fedyshyn Y. Ketela T. (2012). Analysis of early C2C12 myogenesis identifies stably and differentially expressed transcriptional regulators whose knock-down inhibits myoblast differentiation. Physiol. Genomics 44 (2), 183–197. 10.1152/physiolgenomics.00093.2011 22147266

[B98] Ravnskjaer K. Madiraju A. Montminy M. (2016). Role of the cAMP pathway in glucose and lipid metabolism. Handb. Exp. Pharmacol. 233 (2021), 29–49. 10.1007/164_2015_32 26721678

[B99] Reimand J. Isserlin R. Voisin V. Kucera M. Tannus-Lopes C. Rostamianfar A. (2019). Pathway enrichment analysis and visualization of omics data using g:Profiler, GSEA, Cytoscape and EnrichmentMap. Nat. Protoc. 14 (2), 482–517. 10.1038/s41596-018-0103-9 30664679 PMC6607905

[B100] Ritchie M. E. Phipson B. Wu D. Hu Y. Law C. W. Shi W. (2015). Limma powers differential expression analyses for RNA-sequencing and microarray studies. Nucleic Acids Res. 43 (7), e47. 10.1093/nar/gkv007 25605792 PMC4402510

[B101] Rosvall M. Bergstrom C. T. (2010). Mapping change in large networks. PLoS One 5 (1), e8694. 10.1371/journal.pone.0008694 20111700 PMC2811724

[B102] Salazar V. S. Gamer L. W. Rosen V. (2016). BMP signalling in skeletal development, disease and repair. Nat. Rev. Endocrinol. 12 (4), 203–221. 10.1038/nrendo.2016.12 26893264

[B103] Segales J. Perdiguero E. Munoz-Canoves P. (2016). Regulation of muscle stem cell functions: a focus on the p38 MAPK signaling pathway. Front. Cell Dev. Biol. 4 (2016), 91–105. 10.3389/fcell.2016.00091 27626031 PMC5003838

[B104] Sharma M. McFarlane C. Kambadur R. Kukreti H. Bonala S. Srinivasan S. (2015). Myostatin: expanding horizons. IUBMB Life 67 (8), 589–600. 10.1002/iub.1392 26305594

[B105] Signal B. Gloss B. S. Dinger M. E. (2016). Computational approaches for functional prediction and characterisation of long noncoding RNAs. Trends Genet. 32 (10), 620–637. 10.1016/j.tig.2016.08.004 27592414

[B106] Sirabella D. De Angelis L. Berghella L. (2013). Sources for skeletal muscle repair: from satellite cells to reprogramming. J. Cachexia Sarcopenia Muscle 4 (2), 125–136. 10.1007/s13539-012-0098-y 23314905 PMC3684700

[B107] Soleimani V. D. Yin H. Jahani-Asl A. Ming H. Kockx C. van Ijcken W. (2012). Snail regulates MyoD binding-site occupancy to direct enhancer switching and differentiation-specific transcription in myogenesis. Mol. Cell 47 (3), 457–468. 10.1016/j.molcel.2012.05.046 22771117 PMC4580277

[B108] Spittau B. Dokalis N. Prinz M. (2020). The role of TGFβ signaling in microglia maturation and activation. Trends Immunol. 41 (9), 836–848. 10.1016/j.it.2020.07.003 32741652

[B109] Sui Y. Han Y. Zhao X. Li D. Li G. (2019). Long non-coding RNA Irm enhances myogenic differentiation by interacting with MEF2D. Cell Death Dis. 10 (3), 181–193. 10.1038/s41419-019-1399-2 30792383 PMC6385193

[B110] Sun F. Liang W. Tang K. Hong M. Qian J. (2019). Profiling the lncRNA-miRNA-mRNA ceRNA network to reveal potential crosstalk between inflammatory bowel disease and colorectal cancer. PeerJ 7, e7451. 10.7717/peerj.7451 31523496 PMC6714963

[B111] Teng X. Chen X. Xue H. Tang Y. Zhang P. Kang Q. (2020). NPInter v4.0: an integrated database of ncRNA interactions. Nucleic Acids Res. 48 (D1), D160-D165–D165. 10.1093/nar/gkz969 31670377 PMC7145607

[B112] Thomson D. M. (2018). The role of AMPK in the regulation of skeletal muscle size, hypertrophy, and regeneration. Int. J. Mol. Sci. 19 (10), 3125–3144. 10.3390/ijms19103125 30314396 PMC6212977

[B113] Thomson D. W. Dinger M. E. (2016). Endogenous microRNA sponges: evidence and controversy. Nat. Rev. Genet. 17 (5), 272–283. 10.1038/nrg.2016.20 27040487

[B114] Tomczak K. K. Marinescu V. D. Ramoni M. F. Sanoudou D. Montanaro F. Han M. (2004). Expression profiling and identification of novel genes involved in myogenic differentiation. FASEB J. 18 (2), 1–23. 10.1096/fj.03-0568fje 14688207

[B115] Tumpel S. Rudolph K. L. (2019). Quiescence: good and bad of stem cell aging. Trends Cell Biol. 29 (8), 672–685. 10.1016/j.tcb.2019.05.002 31248787

[B116] Vico L. Hargens A. (2018). Skeletal changes during and after spaceflight. Nat. Rev. Rheumatol. 14 (4), 229–245. 10.1038/nrrheum.2018.37 29559713

[B117] von Haehling S. Ebner N. Dos Santos M. R. Springer J. Anker S. D. (2017). Muscle wasting and cachexia in heart failure: mechanisms and therapies. Nat. Rev. Cardiol. 14 (6), 323–341. 10.1038/nrcardio.2017.51 28436486

[B118] Wackerhage H. Del Re D. P. Judson R. N. Sudol M. Sadoshima J. (2014). The Hippo signal transduction network in skeletal and cardiac muscle. Sci. Signal 7 (337), re4. 10.1126/scisignal.2005096 25097035

[B119] Wang S. Jin J. Xu Z. Zuo B. (2019b). Functions and regulatory mechanisms of lncRNAs in skeletal myogenesis, muscle disease and meat production. Cells 8 (9), 1107–1125. 10.3390/cells8091107 31546877 PMC6769631

[B120] Wang S. Zuo H. Jin J. Lv W. Xu Z. Fan Y. (2019a). Long noncoding RNA Neat1 modulates myogenesis by recruiting Ezh2. Cell Death Dis. 10 (7), 505–519. 10.1038/s41419-019-1742-7 31243262 PMC6594961

[B121] Wang W. Min L. Qiu X. Wu X. Liu C. Ma J. (2021). Biological function of long non-coding RNA (LncRNA) xist. Front. Cell Dev. Biol. 9, 645647–645673. 10.3389/fcell.2021.645647 34178980 PMC8222981

[B122] Wang X. H. Mitch W. E. (2014). Mechanisms of muscle wasting in chronic kidney disease. Nat. Rev. Nephrol. 10 (9), 504–516. 10.1038/nrneph.2014.112 24981816 PMC4269363

[B123] Watt K. I. Goodman C. A. Hornberger T. A. Gregorevic P. (2018). The Hippo signaling pathway in the regulation of skeletal muscle mass and function. Exerc Sport Sci. Rev. 46 (2), 92–96. 10.1249/jes.0000000000000142 29346163 PMC6319272

[B124] Wilson B. G. Wang X. Shen X. McKenna E. S. Lemieux M. E. Cho Y. J. (2010). Epigenetic antagonism between polycomb and SWI/SNF complexes during oncogenic transformation. Cancer Cell 18 (4), 316–328. 10.1016/j.ccr.2010.09.006 20951942 PMC2957473

[B125] Winkle M. El-Daly S. M. Fabbri M. Calin G. A. (2021). Noncoding RNA therapeutics - challenges and potential solutions. Nat. Rev. Drug Discov. 20 (8), 629–651. 10.1038/s41573-021-00219-z 34145432 PMC8212082

[B126] Wu N. Zhang X. Bao Y. Yu H. Jia D. Ma C. (2019). Down‐regulation of GAS5 ameliorates myocardial ischaemia/reperfusion injury via the miR‐335/ROCK1/AKT/GSK‐3β axis. J. Cell Mol. Med. 23 (12), 8420–8431. 10.1111/jcmm.14724 31625671 PMC6850918

[B127] Wu T. Hu E. Xu S. Chen M. Guo P. Dai Z. (2021). clusterProfiler 4.0: a universal enrichment tool for interpreting omics data. Innov. (N Y) 2 (3), 100141. 10.1016/j.xinn.2021.100141 PMC8454663 34557778

[B128] Wust S. Drose S. Heidler J. Wittig I. Klockner I. Franko A. (2018). Metabolic maturation during muscle stem cell differentiation is achieved by miR-1/133a-mediated inhibition of the dlk1-dio3 mega gene cluster. Cell Metab. 27 (5), 1026–1039.e6. 10.1016/j.cmet.2018.02.022 29606596

[B129] Xi Y. Shen W. Jin C. Wang L. Yu B. (2020). PVT1 promotes the proliferation and migration of non-small cell lung cancer via regulating miR-148/RAB34 signal Axis. Onco Targets Ther. 13, 1819–1832. 10.2147/ott.s222898 32184617 PMC7054901

[B130] Xiong F. Wei W. P. Liu Y. B. Wang Y. Zhang H. Y. Liu R. (2021). Long noncoding RNA XIST enhances cerebral ischemia-reperfusion injury by regulating miR-486-5p and GAB2. Eur. Rev. Med. Pharmacol. Sci. 25 (4), 2013–2020. 10.26355/eurrev_202102_25103 33660813

[B131] Yao R. W. Wang Y. Chen L. L. (2019). Cellular functions of long noncoding RNAs. Nat. Cell Biol. 21 (5), 542–551. 10.1038/s41556-019-0311-8 31048766

[B132] Yu G. He Q. Y. (2016). ReactomePA: an R/Bioconductor package for reactome pathway analysis and visualization. Mol. Biosyst. 12 (2), 477–479. 10.1039/c5mb00663e 26661513

[B133] Yu X. Zhang Y. Li T. Ma Z. Jia H. Chen Q. (2017). Long non-coding RNA Linc-RAM enhances myogenic differentiation by interacting with MyoD. Nat. Commun. 8, 14016–14027. 10.1038/ncomms14016 28091529 PMC5241866

[B134] Zhang J. Zhao J. Dahan P. Lu V. Zhang C. Li H. (2018a). Metabolism in pluripotent stem cells and early mammalian development. Cell Metab. 27 (2), 332–338. 10.1016/j.cmet.2018.01.008 29414683

[B135] Zhang Z. K. Li J. Guan D. Liang C. Zhuo Z. Liu J. (2018b). A newly identified lncRNA MAR1 acts as a miR-487b sponge to promote skeletal muscle differentiation and regeneration. J. Cachexia Sarcopenia Muscle 9 (3), 613–626. 10.1002/jcsm.12281 29512357 PMC5989759

[B136] Zhang Z. K. Li J. Guan D. Liang C. Zhuo Z. Liu J. (2018c). Long noncoding RNA lncMUMA reverses established skeletal muscle atrophy following mechanical unloading. Mol. Ther. 26 (11), 2669–2680. 10.1016/j.ymthe.2018.09.014 30415659 PMC6225098

[B137] Zhao Y. Chen M. Lian D. Li Y. Li Y. Wang J. (2019). Non-coding RNA regulates the myogenesis of skeletal muscle satellite cells, injury repair and diseases. Cells 8 (9), 988–993. 10.3390/cells8090988 31461973 PMC6769629

[B138] Zhou L. Sun K. Zhao Y. Zhang S. Wang X. Li Y. (2015). Linc-YY1 promotes myogenic differentiation and muscle regeneration through an interaction with the transcription factor YY1. Nat. Commun. 6 (11), 10026. 10.1038/ncomms10026 26658965

[B139] Zhu M. Liu J. Xiao J. Yang L. Cai M. Shen H. (2017). Lnc-mg is a long non-coding RNA that promotes myogenesis. Nat. Commun. 8, 14718–14728. 10.1038/ncomms14718 28281528 PMC5353601

[B140] Zhu Q. Liang F. Cai S. Luo X. Duo T. Liang Z. (2021). KDM4A regulates myogenesis by demethylating H3K9me3 of myogenic regulatory factors. Cell Death Dis. 12 (6), 514–530. 10.1038/s41419-021-03799-1 34011940 PMC8134519

