# Supplementary material for: Developing a ceRNA-based lncRNA-miRNA-mRNA regulatory network to uncover roles in skeletal muscle development
Source: Front Bioinform. 2025 Jan 15;4:1494717. doi: 10.3389/fbinf.2024.1494717 (PMC11774864; doi:10.3389/fbinf.2024.1494717)
Supplement: Supplementary file 1 [file Supplementaryfile1.pdf]

Supplementary Materials for

Developing a ceRNA-based lncRNA-miRNA-mRNA regulatory network to uncover roles in skeletal muscle development

This PDF file includes:

Figure S1 to S5

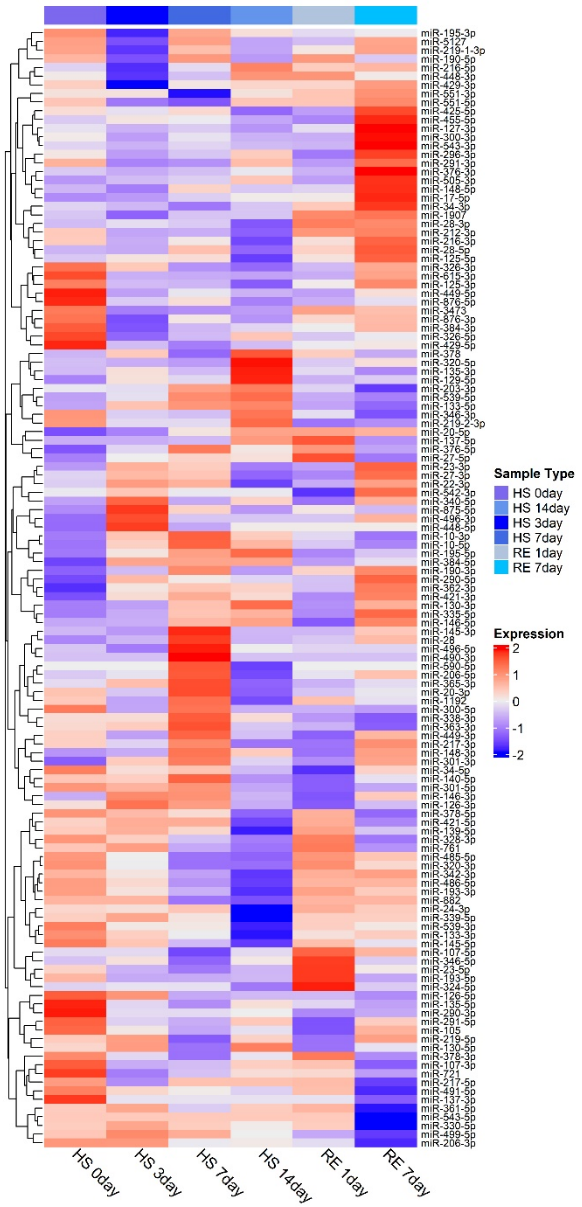

Figure S1 The 129 candidate miRNAs were differentially expressed in miRNAs expression datasets of hindlimb suspension (HS) simulated micrograve. The heat map showing the differentially expressed miRNA during HS 0day, HS 3day, HS 7day HS 14day, RE 1day and RE 14day. HS, hindlimb suspension; RE, Recovery exercise.

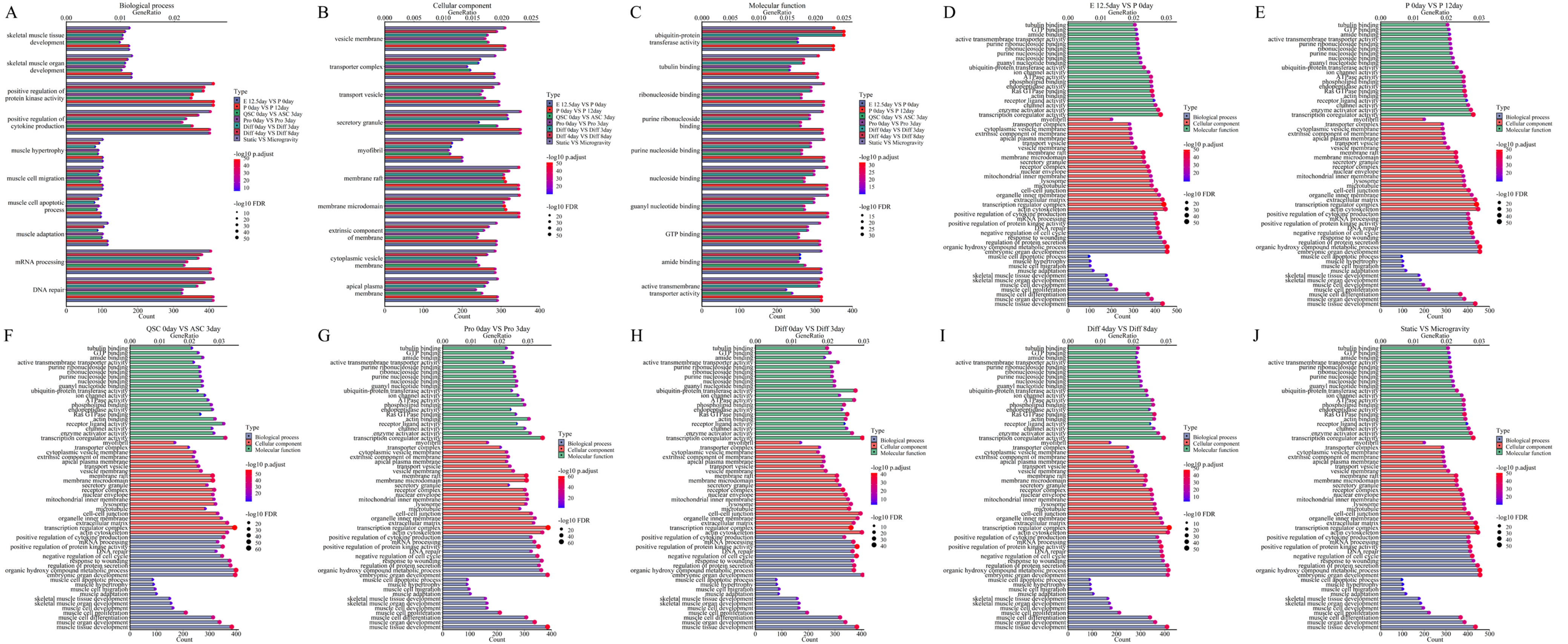

Figure S2 GO enrichment analysis for the lncRNA-miRNA-mRNA ceRNA regulatory network of various stages of skeletal muscle development. (A) The bubble pattern and bar chart show the 10 randomly chosen biological process with Generation and gene count. (B) The bubble pattern and bar chart display the 10 randomly chosen cellular component with generation and gene count. (C) The bubble pattern and bar chart display the 10 randomly chosen molecular function with Generation and gene count. (D) The bubble pattern and bar chart show the 60 randomly chosen GO enrichment pathways with GeneRation and gene count during Embryonic 12.5day VS Postnatal 0day. (E) The bubble pattern and bar chart show the 60 randomly chosen GO enrichment pathways with GeneRation and gene count during Postnatal 0day VS Postnatal 12day. (F) The bubble pattern and bar chart show the 60 randomly chosen GO enrichment pathways with GeneRation and gene count during QSC 0day VS ASC 3day. (G) The bubble pattern and bar chart show the 60 randomly chosen GO enrichment pathways with GeneRation and gene count during Proliferation 0day VS Proliferation 3day. (H) The bubble pattern and bar chart show the 60 randomly chosen GO enrichment pathways with GeneRation and gene count during Differentiation 0day VS Differentiation 3day. (I) The bubble pattern and bar chart show the 60 randomly chosen GO enrichment pathways with GeneRation and gene count during Differentiation 4day VS Differentiation 8day. (J) The bubble pattern and bar chart show the 60 randomly chosen GO enrichment pathways with GeneRation and gene count during static VS microgravity. E, Embryonic; P, Postnatal; QSC, Quiescent satellite cells; ASC, Activated satellite cells; Pro, Proliferation; Diff, Differentiation.

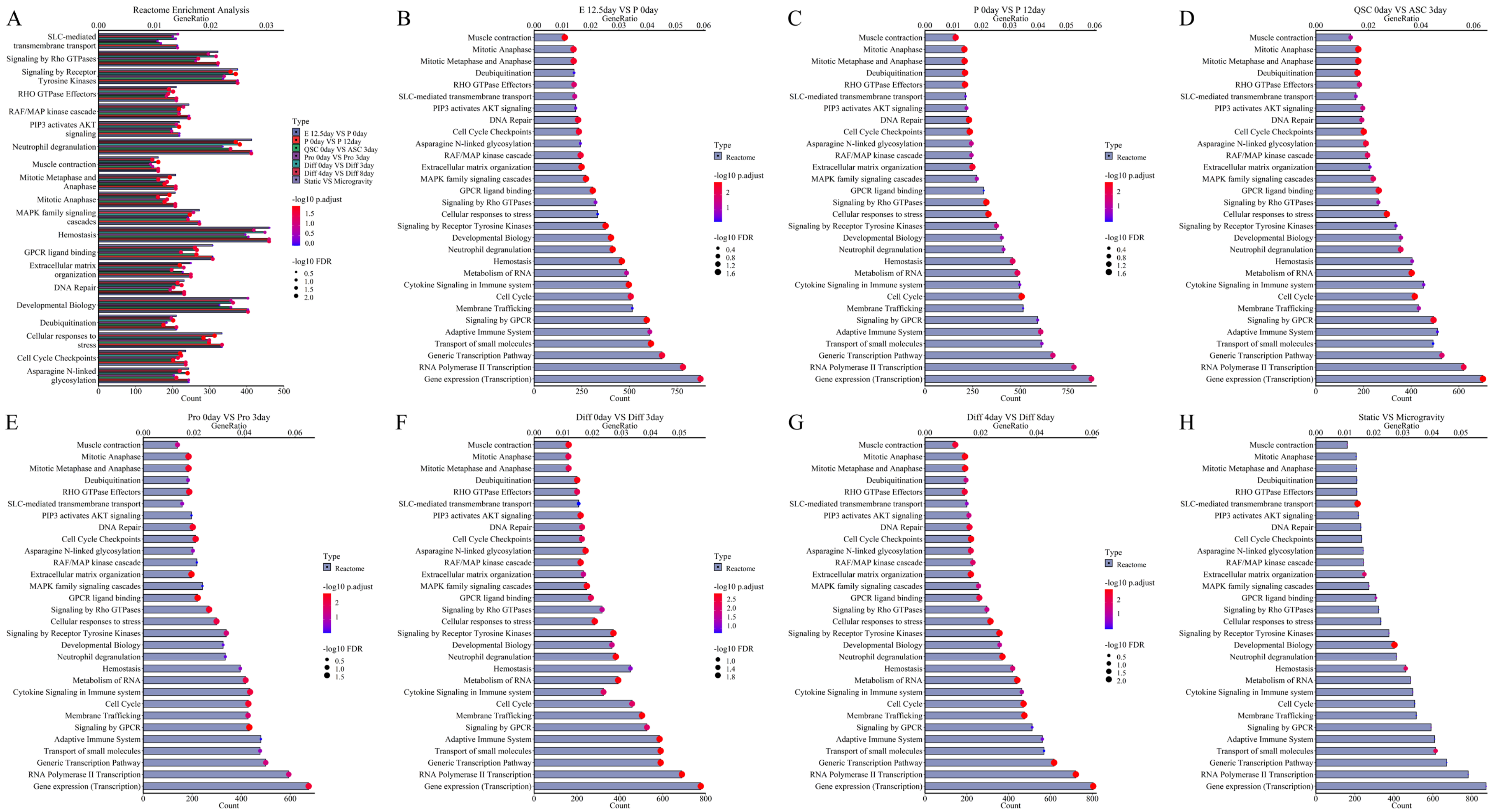

Figure S3 Reactome enrichment analysis for the lncRNA-miRNA-mRNA ceRNA regulatory network of various stages of skeletal muscle development. (A) The bubble pattern and bar chart show the 20 randomly chosen signaling pathway with GeneRatio and gene count. (B) The bubble pattern and bar chart show the 30 randomly chosen Reactome enrichment pathways with GeneRation and gene count during Embryonic 12.5day VS Postnatal 0day. (C) The bubble pattern and bar chart show the 30 randomly chosen Reactome enrichment pathways with GeneRation and gene count during Postnatal 0day VS Postnatal 12day. (D) The bubble pattern and bar chart show the 30 randomly chosen Reactome enrichment pathways with GeneRation and gene count during QSC 0day VS ASC 3day. (E) The bubble pattern and bar chart show the 30 randomly chosen Reactome enrichment pathways with GeneRation and gene count during Proliferation 0day VS Proliferation 3day. (F) The bubble pattern and bar chart show the 30 randomly chosen Reactome enrichment pathways with GeneRation and gene count during Differentiation 0day VS Differentiation 3day. (G) The bubble pattern and bar chart show the 30 randomly chosen Reactome enrichment pathways with GeneRation and gene count during Differentiation 4day VS Differentiation 8day. (H) The bubble pattern and bar chart show the 30 randomly chosen Reactome enrichment pathways with GeneRation and gene count during static VS microgravity. E, Embryonic; P, Postnatal; QSC, Quiescent satellite cells; ASC, Activated satellite cells; Pro, Proliferation; Diff, Differentiation.

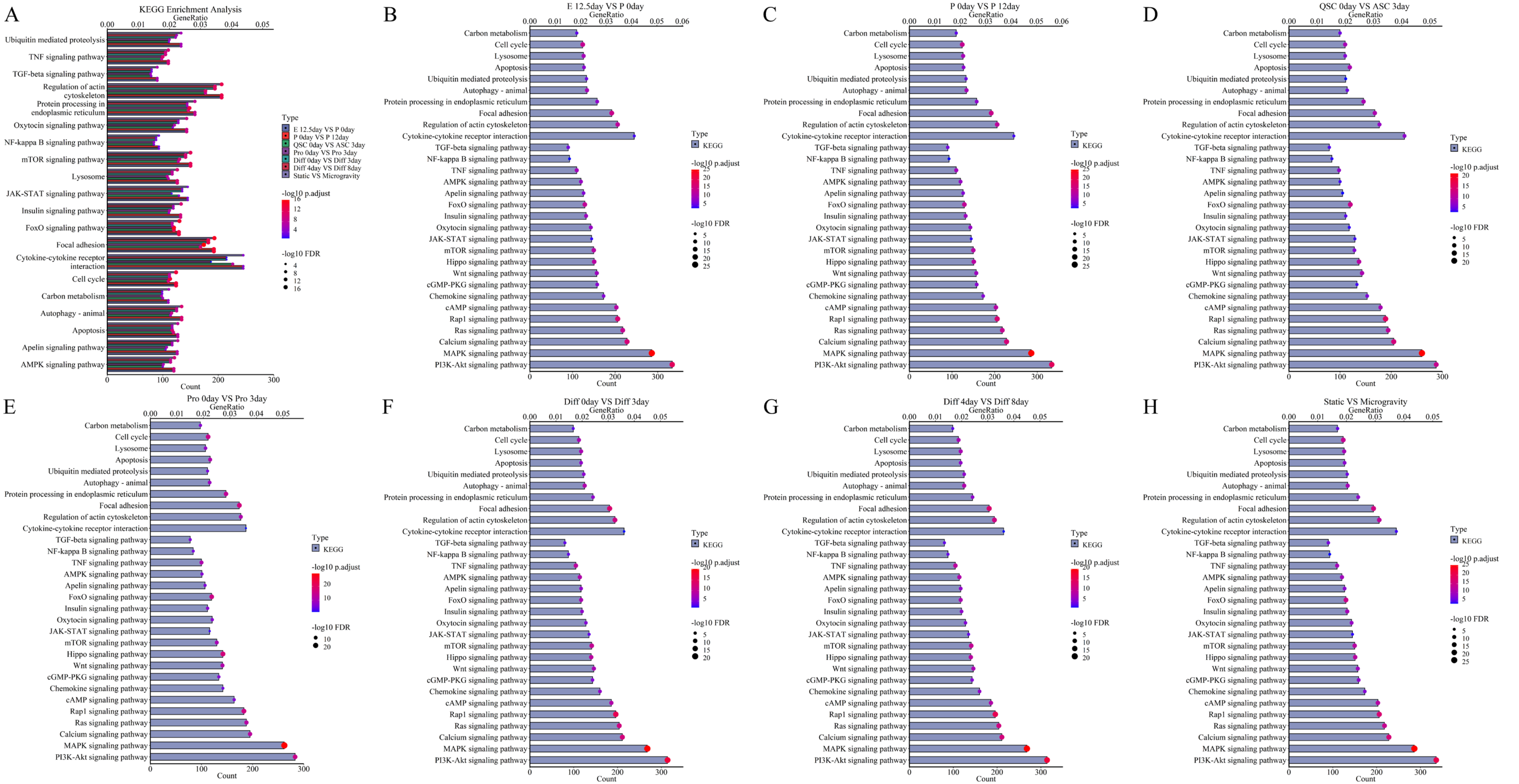

Figure S4 KEGG enrichment analysis for the lncRNA-miRNA-mRNA ceRNA regulatory network of various stages of skeletal muscle development. (A) The bubble pattern and bar chart show the 20 randomly chosen signaling pathway with GeneRatio and gene count. (B) The bubble pattern and bar chart show the 30 randomly chosen KEGG enrichment pathways with GeneRation and gene count during Embryonic 12.5day VS Postnatal 0day. (C) The bubble pattern and bar chart show the 30 randomly chosen KEGG enrichment pathways with GeneRation and gene count during Postnatal 0day VS Postnatal 12day. (D) The bubble pattern and bar chart show the 30 randomly chosen KEGG enrichment pathways with GeneRation and gene count during QSC 0day VS ASC 3day. (E) The bubble pattern and bar chart show the 30 randomly chosen KEGG enrichment pathways with GeneRation and gene count during Proliferation 0day VS Proliferation 3day. (F) The bubble pattern and bar chart show the 30 randomly chosen KEGG enrichment pathways with GeneRation and gene count during Differentiation 0day VS Differentiation 3day. (G) The bubble pattern and bar chart show the 30 randomly chosen KEGG enrichment pathways with GeneRation and gene count during Differentiation 4day VS Differentiation 8day. (H) The bubble pattern and bar chart show the 30 randomly chosen KEGG enrichment pathways with GeneRation and gene count during static VS microgravity. E, Embryonic; P, Postnatal; QSC, Quiescent satellite cells; ASC, Activated satellite cells; Pro, Proliferation; Diff, Differentiation.

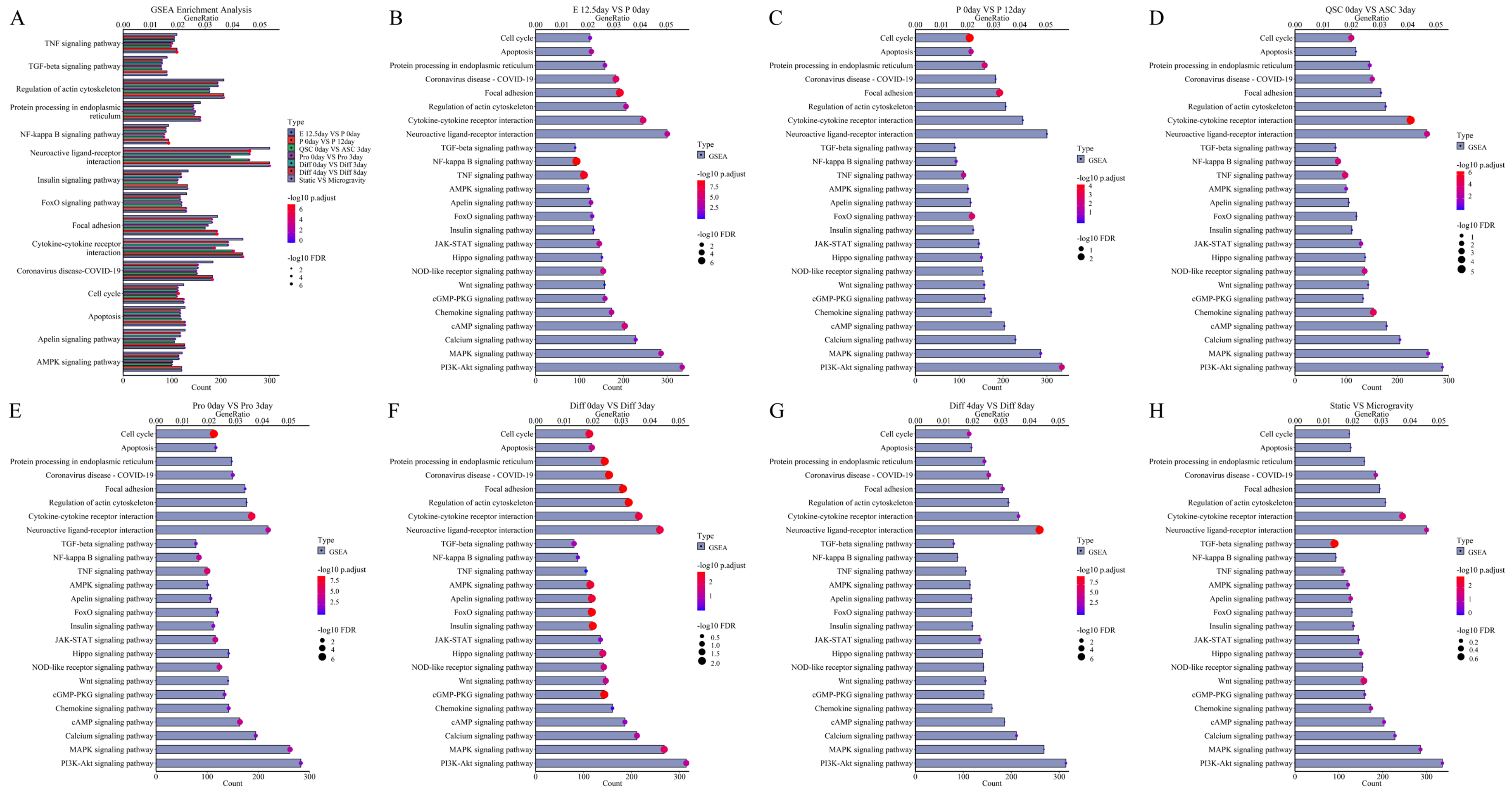

Figure S5 continue

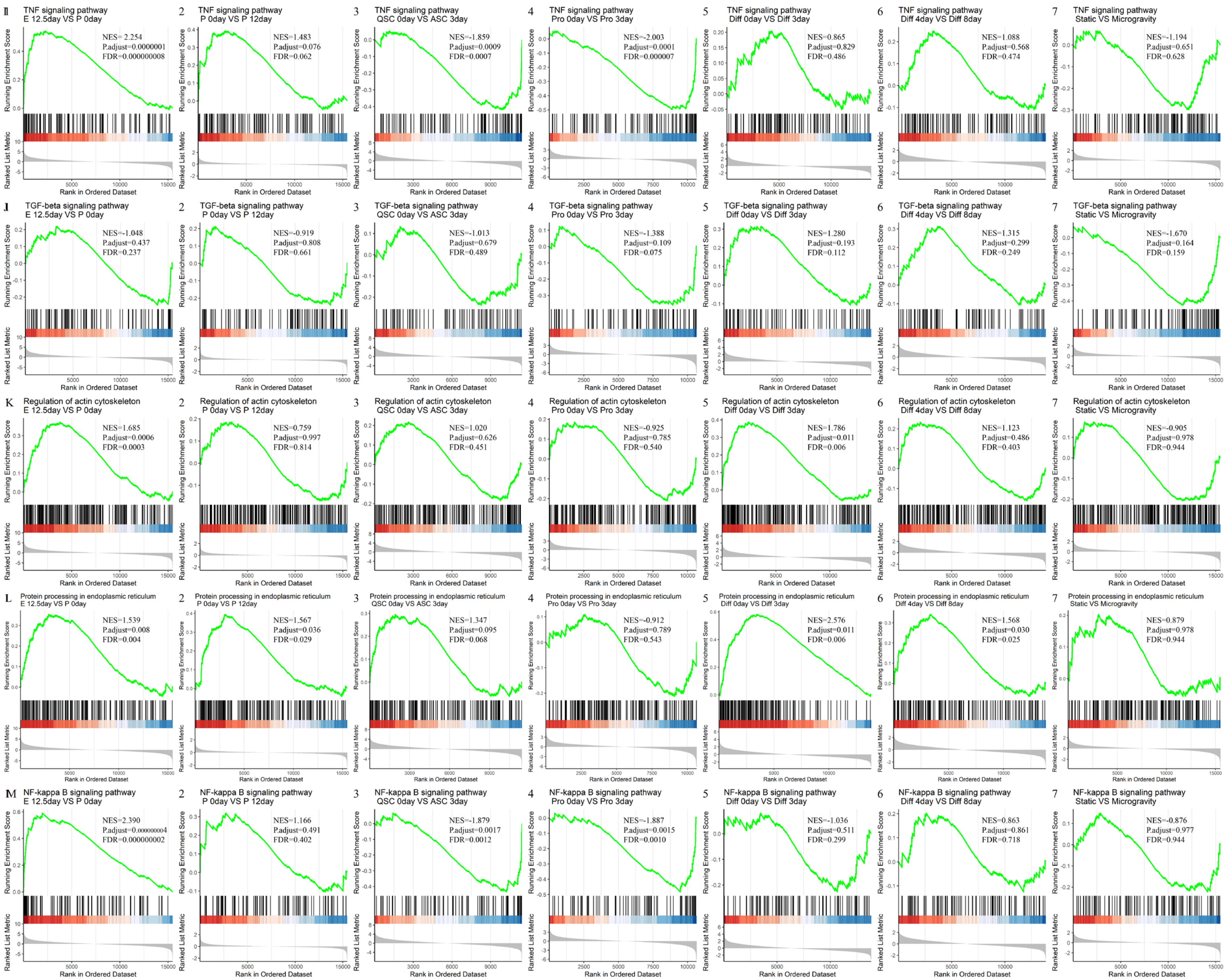

Figure S5 continue

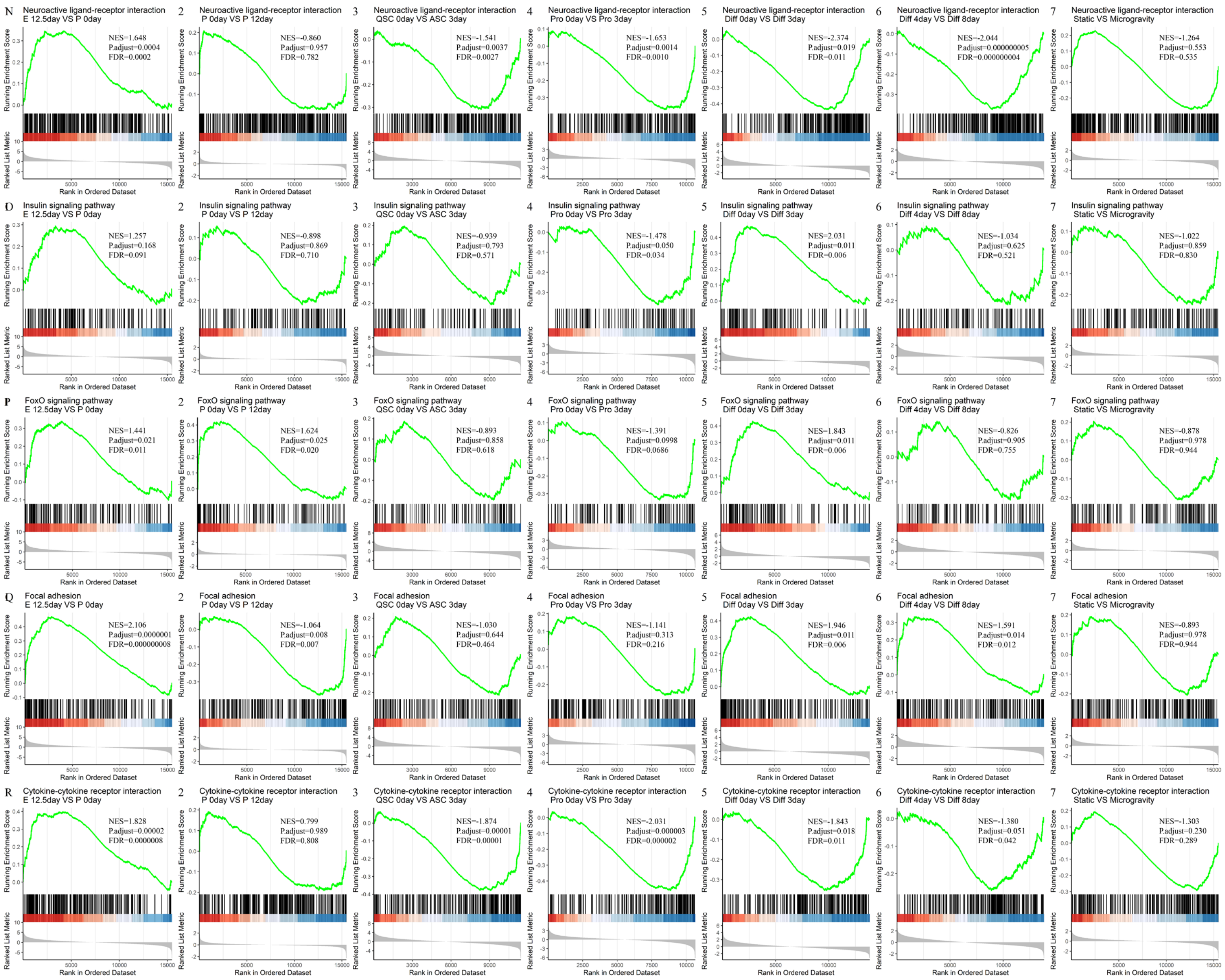

Figure S5 continue

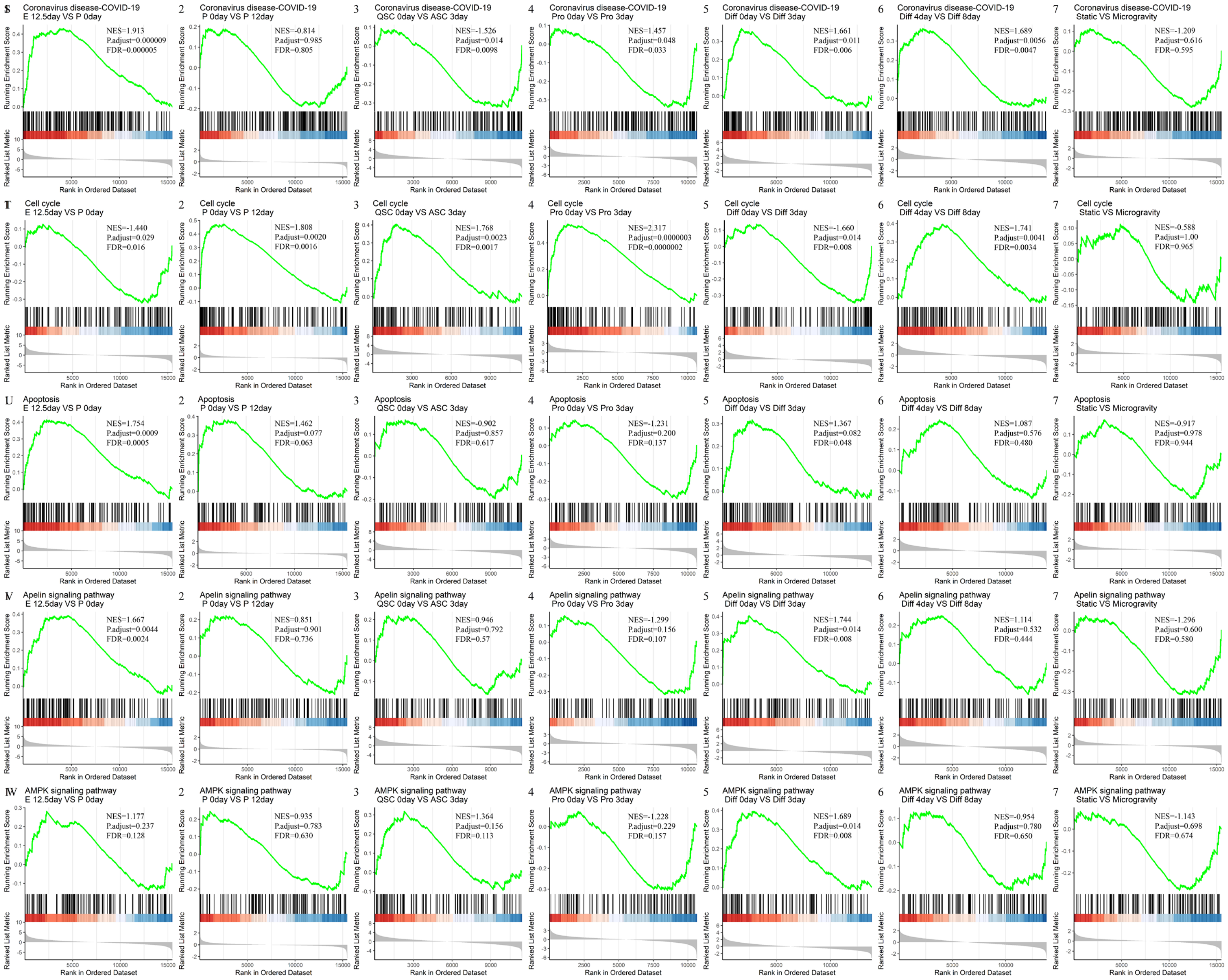

Figure S5 continue

Figure S5 GSEA enrichment analysis for the lncRNA-miRNA-mRNA ceRNA regulatory network of various stages of skeletal muscle development. (A) The bubble pattern and bar chart show the 15 randomly chosen signaling pathway with GeneRatio and gene count. (B) The bubble pattern and bar chart show the 25 randomly chosen GSEA enrichment pathways with GeneRation and gene count during Embryonic 12.5day VS Postnatal 0day. (C) The bubble pattern and bar chart show the 25 randomly chosen GSEA enrichment pathways with GeneRation and gene count during Postnatal 0day VS Postnatal 12day. (D) The bubble pattern and bar chart show the 25 randomly chosen GSEA enrichment pathways with GeneRation and gene count during QSC 0day VS ASC 3day. (E) The bubble pattern and bar chart show the 25 randomly chosen GSEA enrichment pathways with GeneRation and gene count during Proliferation 0day VS Proliferation 3day. (F) The bubble pattern and bar chart show the 25 randomly chosen GSEA enrichment pathways with GeneRation and gene count during Differentiation 0day VS Differentiation 3day. (G) The bubble pattern and bar chart show the 25 randomly chosen GSEA enrichment pathways with GeneRation and gene count during Differentiation 4day VS Differentiation 8day. (H) The bubble pattern and bar chart show the 25 randomly chosen GSEA enrichment pathways with GeneRation and gene count during static VS microgravity. GSEA is a method that determines whether a set of genes shows differences between two biological states. The normalized enrichment score (NES) reflects the degree to which a gene set is upregulated (positive NES) or downregulated (negative NES). Corresponding p values are indicated. (I) GSEA plots showing enrichment of TNF signaling pathway during skeletal muscle development and microgravity-induced muscle atrophy (waste). (J) GSEA plots showing enrichment of TGF-beta signaling pathway during skeletal muscle development and microgravity-induced muscle atrophy (waste). (K) GSEA plots showing enrichment of Actin cytoskeleton signaling pathway during skeletal muscle development and microgravity-induced muscle atrophy (waste). (L) GSEA plots showing enrichment of Protein processing in endoplasmic reticulum signaling pathway during skeletal muscle development and microgravity-induced muscle atrophy (waste). (M) GSEA plots showing enrichment of NF-kappa B signaling pathway during skeletal muscle development and microgravity-induced muscle atrophy (waste). (N) GSEA plots showing enrichment of Neuroactive ligand-receptor interaction signaling pathway during skeletal muscle development and microgravity-induced muscle atrophy (waste). (O) GSEA plots showing enrichment of Insulin signaling pathway during skeletal muscle development and microgravity-induced muscle atrophy (waste). (P) GSEA plots showing enrichment of FoxO signaling pathway during skeletal muscle development and microgravity-induced muscle atrophy (waste). (Q) GSEA plots showing enrichment of Focal adhesion signaling pathway during skeletal muscle development and microgravity-induced muscle atrophy (waste). (R) GSEA plots showing enrichment of Cytokine-cytokine receptor interaction signaling pathway during skeletal muscle development and microgravity-induced muscle atrophy (waste). (S) GSEA plots showing enrichment of Coronavirus disease-COVID-19 signaling pathway during skeletal muscle development and microgravity-induced muscle atrophy (waste). (T) GSEA plots showing enrichment of Cell cycle signaling pathway during skeletal muscle development and microgravity-induced muscle atrophy (waste). (U) GSEA plots showing enrichment of Apoptosis signaling pathway during skeletal muscle development and microgravity-induced muscle atrophy (waste). (V) GSEA plots showing enrichment of Apelin signaling pathway during skeletal muscle development and microgravity-induced muscle atrophy (waste). (W) GSEA plots showing enrichment of AMPK signaling pathway during skeletal muscle development and microgravity-induced muscle atrophy (waste). Normalized enrichment scores (NESs), FDR and nominal p values (Nom p value), as calculated by GSEA, are provided. Skeletal muscle development stages contain E12.5days VS P 0days, P 0days VS P 12days, QSC 0days VS ASC 3days, Pro 0days VS Pro 3days, Diff 0days VS Diff 3days and Diff 4days VS Diff 8days. Microgravity-induced muscle atrophy (waste) is Static VS Microgravity. E, Embryonic; P, Postnatal; QSC, Quiescent satellite cells; ASC, Activated satellite cells; Pro, Proliferation; Diff, Differentiation.
